# Automatic classification of registered clinical trials towards the Global Burden of Diseases taxonomy of diseases and injuries

**DOI:** 10.1186/s12859-016-1247-7

**Published:** 2016-09-22

**Authors:** Ignacio Atal, Jean-David Zeitoun, Aurélie Névéol, Philippe Ravaud, Raphaël Porcher, Ludovic Trinquart

**Affiliations:** 1 Centre d’Épidémiologie Clinique, Hôpital Hôtel-Dieu, Paris, France; 2 INSERM U1153, Paris, France; 3 Université Paris Descartes, Paris, France; 4 LIMSI, CNRS UPR 3251, Université Paris-Saclay, Orsay, France; 5 Department of Epidemiology, Mailman School of Public Health, Columbia University, New York, NY USA

**Keywords:** Clinical trials, Global burden of diseases, Disease classification, Mapping

## Abstract

**Background:**

Clinical trial registries may allow for producing a global mapping of health research. However, health conditions are not described with standardized taxonomies in registries. Previous work analyzed clinical trial registries to improve the retrieval of relevant clinical trials for patients. However, no previous work has classified clinical trials across diseases using a standardized taxonomy allowing a comparison between global health research and global burden across diseases. We developed a knowledge-based classifier of health conditions studied in registered clinical trials towards categories of diseases and injuries from the Global Burden of Diseases (GBD) 2010 study.

The classifier relies on the UMLS® knowledge source (Unified Medical Language System®) and on heuristic algorithms for parsing data. It maps trial records to a 28-class grouping of the GBD categories by automatically extracting UMLS concepts from text fields and by projecting concepts between medical terminologies. The classifier allows deriving pathways between the clinical trial record and candidate GBD categories using natural language processing and links between knowledge sources, and selects the relevant GBD classification based on rules of prioritization across the pathways found. We compared automatic and manual classifications for an external test set of 2,763 trials. We automatically classified 109,603 interventional trials registered before February 2014 at WHO ICTRP.

**Results:**

In the external test set, the classifier identified the exact GBD categories for 78 % of the trials. It had very good performance for most of the 28 categories, especially “Neoplasms” (sensitivity 97.4 %, specificity 97.5 %). The sensitivity was moderate for trials not relevant to any GBD category (53 %) and low for trials of injuries (16 %). For the 109,603 trials registered at WHO ICTRP, the classifier did not assign any GBD category to 20.5 % of trials while the most common GBD categories were “Neoplasms” (22.8 %) and “Diabetes” (8.9 %).

**Conclusions:**

We developed and validated a knowledge-based classifier allowing for automatically identifying the diseases studied in registered trials by using the taxonomy from the GBD 2010 study. This tool is freely available to the research community and can be used for large-scale public health studies.

**Electronic supplementary material:**

The online version of this article (doi:10.1186/s12859-016-1247-7) contains supplementary material, which is available to authorized users.

## Background

The World Health Organization (WHO) has indicated the pressing need for a comprehensive monitoring of health research and development (R&D) to coordinate limited resources towards reducing the gaps between health research and health needs [1–3]. Mapping the global landscape of health R&D will allow for identifying diseases for which there is too much or too little research at a local level as compared to their burden at the same level [4]. The WHO is developing the Global Observatory on Health R&D and aims at analyzing multiple data sources to quantify the global state of health R&D, including clinical trial registries, publications, product pipelines, patents and grants [3, 5].

Although concerning a particular type of health R&D activity, one source of data, clinical trial registries, is readily available and could be used to rapidly achieve a global mapping [6]. Worldwide, clinical trials are registered in publicly accessible repositories with a common structure of data fields [7]. The WHO gathers 16 registries in the International Clinical Trials Registry Platform (ICTRP), now the largest repository of clinical trials worldwide [8].

However, the diseases studied by clinical trials registered in the WHO ICTRP are not described in trial records by using a standardized taxonomy but rather as free text with considerable heterogeneity. With more than 300,000 clinical trial records in the WHO ICTRP and more than 20,000 new records registered every year, the use of automatic methods for classification is imperative [8, 9]. Natural Language Processing (NLP) allows clinical knowledge representation in standardized formats and is becoming mature enough to be used efficiently for targeted applications [10, 11]. In particular, NLP methods have been developed to face the limitations of the retrieval systems of clinical trial registries such as clinicaltrials.gov. [12, 13] For instance, clinical trial records have been notably analyzed using NLP to provide formal representations of eligibility criteria, or to enrich eligibility criteria with meta-data to improve the retrieval of relevant clinical trials for patients [14–26]. However, none of these studies have analyzed the performance of retrieval of clinical trials across diseases, but rather across features of eligibility criteria (e.g. age, BMI 1 or more complex features) for specific diseases.

Moreover, the health conditions studied in registered clinical trials must be classified by using a taxonomy of diseases that allows for comparisons between the numbers of clinical trials and the actual burden of diseases. A consensual taxonomy over which the evolution of the burden is estimated regionally was developed by the US Institute for Health Metrics and Evaluation for the Global Burden of Diseases (GBD) 2010 study [27, 28]. Previous studies have developed NLP methods to index clinical trial records using Medical Subject Headings (MeSH) [29], and to regroup clinical trials across medical specialties [30]. However, to our knowledge no previous work has classified clinical trials using a taxonomy allowing a comparison between global health research and global burden across diseases.

### Objective

We aimed to develop and validate a method that automatically maps the health conditions studied in registered clinical trials to the taxonomy from the GBD 2010 study. Towards that goal, we relied on Natural Language Processing to analyze the free-text description of health conditions found in clinical trial records, and a standardized knowledge representation of diseases to encode the information extracted from the trial records.

## Methods

We developed a knowledge-based classifier allowing for automatic mapping of the health conditions studied in registered clinical trials to a 28- and 171-class grouping of the taxonomy of diseases and injuries defined by the GBD 2010 study. Our approach did not rely on statistical classification techniques but instead relied on text analysis and exploited the Unified Medical Language System® (UMLS®) as a domain knowledge resource. Specifically, the classification is based on the recognition of medical concepts in the free text description of trials and the mapping of concepts between medical taxonomies. The classifier allows deriving pathways between the clinical trial record and the taxonomy of diseases and injuries from the GBD study based on a succession of mathematical projections (also called normalization or entity linking). Finally, the classifier selects the relevant GBD classification based on rules of prioritization across the pathways found. We measured the classifier performance by comparing the automatic classifications to manual classifications with a large test set of registered clinical trials. Finally, we used the classifier to map the conditions studied by all trials registered at the WHO ICTRP.

### From clinical trial records to the GBD cause list

#### GBD cause list

The GBD cause list is a set of 291 mutually exclusive and collectively exhaustive categories of diseases and injuries [28]. Each category is defined in terms of the codes of the International Classification of Diseases 9th and 10th versions (ICD9 and ICD10) [31]. We used the mapping from the ICD10 to the GBD cause list (Web Table 3 in [27]). Several residual categories, such as “Other infectious diseases”, are made up of ill-defined or residual causes from major disease groups. We excluded these because they are not informative from the perspective of a global analysis of the burden of diseases.

We developed a smaller list of categories by using a formal consensus method. Six experts independently defined a higher-level grouping of diseases and injuries that are sufficiently informative for developing a global mapping of clinical trials across health needs. The resulting list contained 28 categories that accounted for 98.8 % of the global burden in 2010 (Table 1). Moreover, we considered the list of aggregated categories defined by the GBD 2010 study to inform policy makers on the main health problems per country (Web Table 1 in [28]). This grouping contained 171 GBD categories that accounted for 90.6 % of the global burden of disease in 2010 (Additional file 1: Table S1). We report results of the mapping to the 28 categories; results of the mapping to the 171 categories are presented in the Additional file 1.

**Table 1:** Grouping of the Global Burden of Diseases (GBD) cause list in 28 GBD categories

GBD categories	Partition of the GBD cause list
Tuberculosis	Tuberculosis
HIV/AIDS	HIV/AIDS
Diarrhea, lower respiratory infections, meningitis, and other common infectious diseases	Diarrheal diseases; Typhoid and paratyphoid fevers; Lower respiratory infections; Upper respiratory infections; Otitis media: Meningitis; Encephalitis; Diphtheria; Whooping cough; Tetanus; Measles; Varicella
Malaria	Malaria
Neglected tropical diseases excluding malaria	Chagas disease; Leishmaniasis: African trypanosomiasis; Schistosomiasis; Cysticercosis; Echinococcosis; Lymphatic filariasis; Onchocerciasis; Trachoma; Dengue; Yellow fever; Rabies; Food-borne trematodiases; Intestinal nematode infections; Other neglected tropical diseases
Maternal disorders	Maternal hemorrhage; Maternal sepsis; Hypertensive disorders of pregnancy; Obstructed labor; Abortion; Other maternal disorders
Neonatal disorders	Preterm birth complications; Neonatal encephalopathy (birth asphyxia and birth trauma); Sepsis and other infectious disorders of the newborn baby; Other neonatal disorders
Nutritional deficiencies	Protein-energy malnutrition; Iodine deficiency; Vitamin A deficiency; Iron-deficiency anemia; Other nutritional deficiencies
Sexually transmitted diseases excluding HIV	Syphilis; Sexually transmitted chlamydial diseases; Gonococcal infection; Trichomoniasis; Other sexually transmitted diseases
Hepatitis	Acute hepatitis A; Acute hepatitis B; Acute hepatitis C; Acute hepatitis E
Leprosy	Leprosy
Neoplasms	Esophageal cancer; Stomach cancer; Liver cancer; Larynx cancer; Trachea, bronchus, and lung cancers; Breast cancer; Cervical cancer; Uterine cancer; Prostate cancer; Colon and rectum cancers; Mouth cancer; Nasopharynx cancer; Cancer of other part of pharynx and oropharynx; Gallbladder and biliary tract cancer; Pancreatic cancer; Malignant melanoma of skin; Non-melanoma skin cancer; Ovarian cancer; Testicular cancer; Kidney and other urinary organ cancers; Bladder cancer; Brain and nervous system cancers; Thyroid cancer; Hodgkin's disease; Non-Hodgkin lymphoma; Multiple myeloma; Leukemia; Other neoplasms
Cardiovascular and circulatory diseases	Rheumatic heart disease; Ischemic heart disease; Cerebrovascular disease; Hypertensive heart disease; Cardiomyopathy and myocarditis; Atrial fibrillation and flutter; Aortic aneurysm; Peripheral vascular disease; Endocarditis; Other cardiovascular and circulatory diseases
Chronic respiratory diseases	Chronic obstructive pulmonary disease; Pneumoconiosis; Asthma; Interstitial lung disease and pulmonary sarcoidosis; Other chronic respiratory diseases
Cirrhosis of the liver	Cirrhosis of the liver
Digestive diseases (except cirrhosis)	Peptic ulcer disease; Gastritis and duodenitis; Appendicitis; Paralytic ileus and intestinal obstruction without hernia; Inguinal or femoral hernia; Non-infective inflammatory bowel disease; Vascular disorders of intestine; Gall bladder and bile duct disease; Pancreatitis; Other digestive diseases
Neurological disorders	Alzheimer's disease and other dementias; Parkinson's disease; Epilepsy; Multiple sclerosis; Migraine; Tension-type headache; Other neurological disorders
Mental and behavioral disorders	Schizophrenia; Alcohol use disorders; Drug use disorders; Unipolar depressive disorders; Bipolar affective disorder; Anxiety disorders; Eating disorders; Pervasive development disorders; Childhood behavioral disorders; Idiopathic intellectual disability; Other mental and behavioral disorders
Diabetes, urinary diseases and male infertility	Diabetes mellitus; Acute glomerulonephritis; Chronic kidney diseases; Urinary diseases and male infertility
Gynecological diseases	Uterine fibroids; Polycystic ovarian syndrome; Female infertility; Endometriosis; Genital prolapse; Premenstrual syndrome; Other gynecological diseases
Hemoglobinopathies and hemolytic anemias	Hemoglobinopathies and hemolytic anemias; Thalassemias; Sickle cell disorders; G6PD deficiency; Other hemoglobinopathies and hemolytic anemias
Musculoskeletal disorders	Rheumatoid arthritis; Osteoarthritis; Low back and neck pain; Gout; Other muskuloskeletal disorders
Congenital anomalies	Congenital anomalies; Neural tube defects; Congenital heart anomalies; Cleft lip and cleft palate; Down's syndrome; Other chromosomal abnormalities; Other congenital anomalies
Skin and subcutaneous diseases	Eczema; Psoriasis; Cellulitis; Abscess, impetigo, and other bacterial skin diseases; Scabies; Fungal skin diseases; Viral skin diseases; Acne vulgaris; Alopecia areata; Pruritus; Urticaria; Decubitus ulcer; Other skin and subcutaneous diseases
Sense organ diseases	Glaucoma; Cataracts; Macular degeneration; Refraction and accommodation disorders; Other hearing loss; Other vision loss; Other sense organ diseases
Oral disorders	Dental caries; Periodontal disease; Edentulism
Sudden infant death syndrome	Sudden infant death syndrome
Injuries	Transport injuries; Unintentional injuries other than transport injuries; Self-harm and interpersonal violence; Forces of nature, war, and legal intervention
Excluded residual categories	Other infectious diseases; Other endocrine, nutritional, blood, and immune disorders

Grouping of the cause list of diseases and injuries from the Global Burden of Diseases 2010 study in 28 GBD categories, plus the excluded residual categories. This grouping was considered sufficiently informative for a global mapping of health research to a global mapping of health needs

#### Clinical trial records

**Fig. 1:**
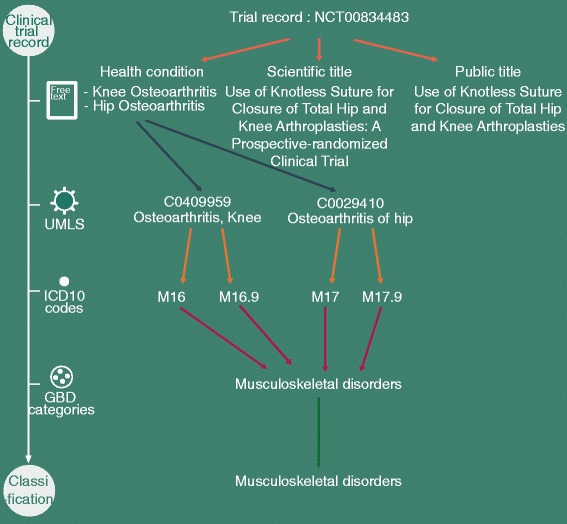
Example of classification of a clinical trial record towards the GBD categories. The classification process is based on text extraction from the trial record, text annotation using UMLS concepts, projection of UMLS concepts to ICD10 codes, projection of ICD10 codes to candidate GBD categories among the 28 GBD categories, and GBD classification based on the candidate GBD categories. In this example, the text annotation involved use of the WSD server for MetaMap, and no expert-based enrichment was needed

In the WHO Trial Registration Dataset, the “Health Condition(s) or Problem(s) studied” field contains a natural language description of the primary condition or problem studied in any clinical trial. Figure 1 shows an example for which the health condition field is “Knee Osteoarthritis” and “Hip Osteoarthritis”. This description is not captured by a coded field, with a standardized taxonomy of diseases, but is rather described in a free-text field. Moreover, the analysis of this free-text field alone may not be sufficient to identify the GBD categories of interest. Numerous health condition fields are empty, have entry errors, correspond to “Healthy volunteers”, or the relevant GBD category may be difficult to identify because of synonymy. Thus, we also considered the “Public Title” and “Scientific Title” fields, which are most likely to bring additional information about the condition studied in the clinical trial and to enrich the mapping.

### Classifier development

Because GBD categories are defined by ICD10 codes, we aimed to classify the text fields according to ICD10 codes. The Unified Medical Language System® (UMLS®), developed at the US National Library of Medicine (NLM), is the most comprehensive metathesaurus to analyze biomedical text in English to date [32]. We based our classifier on established methods using the UMLS knowledge source to automatically annotate trial records with ICD10 codes.

**Fig. 2:**
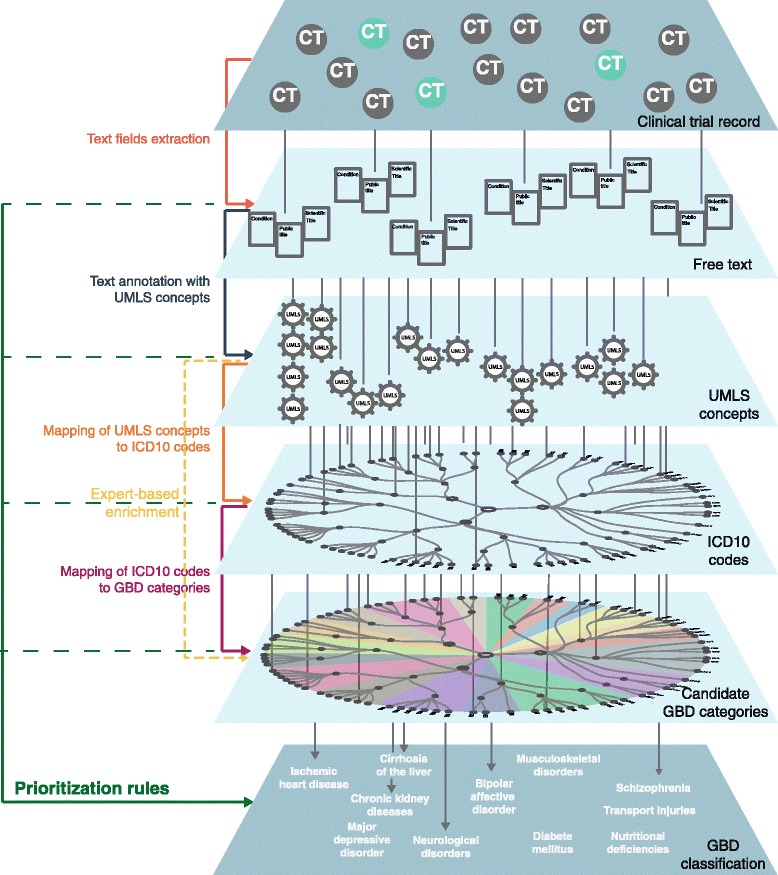
Methodological stages for classification. The classification of clinical trial records has 5 stages. The 4 initial stages allow for deriving pathways from the clinical trial record to candidate GBD categories: annotation of text from the trial record with UMLS concepts by using MetaMap, projection of UMLS concepts to ICD10 codes with IntraMap, projection of ICD10 codes to candidate GBD categories, and expert-based enrichment when automatic pathways are not possible. The fifth stage allows for deriving the GBD classification of the trial based on prioritization rules over the pathways found

Figure 2 illustrates the 5 methodological stages we defined for the classifier (interactive version at http://clinicalepidemio.fr/gbd_graph). The 4 initial stages allow for deriving pathways from the clinical trial record to candidate GBD categories. The 5th stage allows for deriving the GBD classification based on prioritization rules over the pathways found.

#### Free text annotation with concepts from the unified medical language system

We first annotated the text fields (health condition, public title and scientific title) with concepts from the UMLS metathesaurus [32]. The annotation involved use of MetaMap, a tool from the NLM for recognizing UMLS concepts in text [33]. We considered only UMLS concepts corresponding to diseases or injuries (MetaMap implementation in Additional file 1). A Word Sense Disambiguation (WSD) server can be used to select a single UMLS concept when a text is annotated with several UMLS concepts. We developed the classifier with and without using the WSD server. In Fig. 1, the health condition field was annotated with the concepts “Osteoarthritis, Knee” (C0409959) and “Osteoarthritis of hip” (C0029410).

#### Mapping of UMLS concepts to ICD10 codes

Each UMLS concept was then projected to one or several ICD10 codes. The projection involved a semantic-based approach to connect different terminologies present in the UMLS database, namely the Restrict-to-ICD10 algorithm, as implemented in the IntraMap program (IntraMap implementation in Additional file 1) [34]. In the example from Fig. 1, the concept “Osteoarthritis, Knee” was projected to the ICD10 codes “Coxarthrosis [arthrosis of hip]” and “Coxarthrosis, unspecified”.

#### Mapping of ICD10 codes to candidate GBD categories

The resulting ICD10 codes were then projected to one or several candidate GBD categories. ICD10 codes could correspond to three- and four-character ICD10 codes (e.g. M16 and M16.9 in the example from Fig. 1), or to blocks of three- and four-character ICD10 codes (e.g. F30–F39.9). Three- and four-character ICD10 codes were projected to a GBD category only if it was totally included in an unique GBD category. For instance, the ICD10 code P37 could not be projected to a GBD category as P37.0 was included in the GBD category “Tuberculosis”, and P37.3 was included in the GBD category “Neglected tropical diseases excluding malaria”. Blocks of ICD10 codes were split into a list of three- and four-character ICD10 codes (e.g. F30–F39.9 was split into F30, F31, …, F39.9). The block of ICD10 codes was projected to the GBD category(ies) corresponding to the individual projections of the three- and four-character ICD10 codes. In the example from Fig. 1, the ICD10 codes were projected to the GBD category “Musculoskeletal disorders”.

#### Expert-based enrichment

Some UMLS concepts were not mapped to any candidate GBD category. We manually reviewed those UMLS concepts appearing in more than 10 clinical trials registered at the WHO ICTRP database by February 2014 and projected them to candidate GBD categories when relevant. We manually reviewed 503 UMLS concepts, among which 62 could be projected to candidate GBD categories (Additional file 1: Datasets S1 and S2). We developed the classifier with and without the expert-based enrichment.

#### Prioritization rules for GBD classification

For each trial, the previous stages resulted in several pathways from the health condition, the public title and the scientific title fields to multiple candidate GBD categories, respectively. These pathways may pass through several UMLS concepts and ICD10 codes. We developed rules of prioritization to define the GBD classification.

We gave priority to pathways issued from the health condition field because, by definition, it contains the information about the health condition(s) studied in the clinical trial. We also gave priority to candidate GBD categories for which the trial record was consistently projected by several pathways versus candidate GBD categories reached by isolated pathways. This rule aims at discarding candidate GBD categories that may appear by noise (Prioritization rules in Additional file 1). We developed the classifier with and without the rule of giving priority to the health condition field. In the example from Fig. 1, all the pathways from the trial record arrived at the same GBD category, “Musculoskeletal disorders”.

Note that for some trials, the classifier may not find any GBD category. These trials may study health conditions corresponding to residual categories or health conditions not relevant for the GBD 2010 study (eg, pain management). These trials were classified as “No GBD” category trials.

### External validation

We compared the automatic classification to a manual classification (considered the gold standard) for a large test set of registered clinical trials. We measured the performance of 8 versions of the classifier, corresponding to the combinations of using or not the WSD server, using or not the expert-based enrichment, and giving or not priority to the health condition field.

#### Clinical trial data used in our study

The test set included data from 3 different sources. First, we used data from the Epidemiological Study of Randomized Trials, which selected all primary publications of clinical trials published in December 2012 and indexed in PubMed by November 2013 [35]. Among the 1,351 publications, we identified 519 trials registered at the WHO ICTRP. Two independent physicians manually classified each publication according to GBD categories. Second, we used data from a WHO study that extracted a random 5 % sample of clinical trials of interventions registered in the ICTRP by August 2012 [36]. One physician classified 2,381 trial records with GBD categories according to Table C3 in [37], with consensus with a second physician in case of ambiguity. We identified 1,271 trial records for which the classification could be unambiguously mapped to our grouping of GBD categories. Finally, we used data from an ongoing study from our team that involves 973 clinical trials of cancer registered at ICTRP before June 2015. One physician classified each record according to GBD categories, with consensus with a second physician in case of doubt. In total we included 2,763 trials in the external test set (Test set of clinical trials in Additional file 1).

#### Evaluation metrics

We assessed the performance of the classifier by measuring the proportion of trials for which the automatic classification corresponded exactly to the gold standard (exact-matching). We evaluated the exact-matching over trials concerning a unique GBD category, two or more GBD categories and no GBD categories. We computed the overall exact-matching separately for each source of data. We chose the best version of the classifier according to the overall exact-matching proportion. For the best version of the classifier, we evaluated the sensitivities, specificities and positive predictive values for each GBD category. The positive predictive value gives the probability that the trial truly concerned the GBD category identified. If the sensitivity is high for a GBD category, a negative result rules out the category; if the specificity is high, a positive result rules in the category. We derived the positive and negative likelihood ratios (LR+ and LR-); we considered that the classifier reliably identified GBD categories when LR+ > 10 (ruling in the disease), and LR- < 0.1 (ruling out the disease). We computed the weighted average of the sensitivities and specificities across categories.

Lastly, to put the performance measures of the knowledge-based classifier into context, we compared them to a baseline using a simple method of classification. The baseline did not used the UMLS knowledge source, but a clinical trial record was classified to a GBD category if at least one of the disease names defining that GBD category appeared verbatim in the condition field, the public or scientific titles, separately, or in at least one of these three text fields (for disease names used see Table 1 and Web Table 1 in [28]).

### Classification of all clinical trials registered in the WHO ICTRP database

We downloaded all trial records available at the WHO ICTRP by February 1, 2014. We classified all interventional trials initiated between 2006 and 2012 by applying the best-performing version of the classifier. We evaluated the total number of trials mapped to each GBD category.

### Research reproducibility

The classifier was coded by using R 3.2.2 (R Development Core Team, Vienna, Austria). The programs of the classifier is publicly available for the research community to use at the open source platform github (github.com/iatal/trial_gbd). It includes all the codes underlying the classification of clinical trial records downloaded at the WHO ICTRP or at clinicaltrials.gov websites towards the 28- or 171-class grouping of GBD categories. In addition, an online interface to optimize manual classification of clinical trials records registered at the WHO ICTRP is available at (http://www.clinicalepidemio.fr/gbd_study_who/). Finally, the classification using the best-performing version of the classifier is provided for all interventional trials registered at WHO ICTRP (*N* = 109,603 trials by February 2014, Additional file 2).

## Results

Among 2,763 trials in the external test set, 2,328 (84.3 %) concerned a single GBD category, 28 (1.0 %) 2 or more GBD categories, and 407 (14.7 %) residual categories or health conditions not relevant in the GBD 2010 study. Many clinical trials studied “Neoplasms” (958 trials), followed by “Diabetes, urinary diseases and male infertility” (242 trials) and “Cardiovascular and circulatory diseases” (235 trials) (Table 2 and Additional file 1: Table S2).

**Table 2:** Distribution of the external test set (*n* = 2,763 trials) across the 28-class grouping of the GBD cause list, performance of the best performing version of the classifier in the external test set, and projection of all trials in the WHO ICTRP database (*n* = 109,603)

	External test set						WHO ICTRP
GBD categories	No. trials	Sen (%)	Spe (%)	PV+ (%)	LR+	LR-	No. trials (%)
Neoplasms	958	97.4 [96.7-97.7]	97.5 [97.0-97.7]	95.3 [94.4-95.8]	38.2 [28.7-50.8]	0.03 [0.02-0.04]	25,004 (22.8)
Diabetes, urinary diseases and male infertility	242	81.0 [78.0-83.0]	97.4 [97.0-97.7]	75.1 [72.1-77.4]	31.4 [24.5-40.2]	0.20 [0.15-0.25]	9,749 (8.9)
Cardiovascular and circulatory diseases	235	75.7 [72.5-78.1]	97.6 [97.2-97.9]	74.8 [71.6-77.2]	31.9 [24.6-41.4]	0.25 [0.20-0.31]	8,906 (8.1)
Mental and behavioral disorders	143	93.7 [90.5-94.7]	98.7 [98.4-98.9]	80.2 [76.5-82.6]	74.4 [52.9-104.7]	0.06 [0.03-0.12]	7,609 (6.9)
Musculoskeletal disorders	113	88.5 [84.2-90.3]	98.5 [98.2-98.7]	71.4 [67.1-74.6]	58.6 [42.8-80.3]	0.12 [0.07-0.19]	6,112 (5.6)
HIV/AIDS	97	88.7 [83.9-90.4]	99.7 [99.6-99.8]	92.5 [88.0-93.6]	337.7 [160.6-710.0]	0.11 [0.07-0.20]	2,295 (2.1)
Neurological disorders	93	84.9 [79.9-87.3]	98.5 [98.2-98.7]	66.4 [61.6-70.1]	56.7 [41.2-78.0]	0.15 [0.09-0.25]	6,355 (5.8)
Chronic respiratory diseases	81	93.8 [89.0-94.6]	99.4 [99.1-99.5]	81.7 [76.5-84.4]	148.0 [91.9-238.5]	0.06 [0.03-0.15]	4,104 (3.7)
Sense organ diseases	56	92.9 [86.5-93.7]	98.5 [98.2-98.7]	56.5 [51.2-61.3]	62.8 [45.8-86.2]	0.07 [0.03-0.19]	3,461 (3.2)
Injuries	56	16.1 [13.4-23.1]	99.5 [99.3-99.6]	39.1 [31.2-50.1]	31.1 [14.0-68.8]	0.07 [0.03-0.19]	655 (0.6)
Diarrhea, lower respiratory infections, meningitis, and other common infectious diseases	49	81.6 [73.9-84.8]	99.2 [99.0-99.3]	65.6 [58.7-70.6]	105.5 [67.5-164.8]	0.19 [0.10-0.33]	3,200 (2.9)
Maternal disorders	43	39.5 [33.2-47.6]	99.8 [99.7-99.8]	77.3 [64.7-81.7]	215.1 [83.1-556.4]	0.61 [0.48-0.77]	602 (0.5)
Digestive diseases (except cirrhosis)	32	75.0 [65.0-79.7]	99.0 [98.7-99.1]	46.2 [39.7-53.1]	73.2 [48.1-111.3]	0.25 [0.14-0.46]	4,454 (4.1)
Cirrhosis of the liver	23	82.6 [70.2-85.6]	99.4 [99.2-99.5]	52.8 [44.6-60.4]	133.1 [80.0-221.6]	0.17 [0.07-0.43]	1,412 (1.3)
Congenital anomalies	23	95.7 [78.1-99.9]	98.8 [98.5-98.9]	39.3 [33.7-46.3]	77.1 [54.6-108.9]	0.04 [0.01-0.30]	1,947 (1.8)
Skin and subcutaneous diseases	22	81.8 [69.1-85.1]	99.1 [98.9-99.2]	42.9 [36.1-50.8]	93.4 [59.9-145.7]	0.18 [0.08-0.45]	3,652 (3.3)
Hepatitis	17	82.4 [67.5-85.3]	99.9 [99.7-99.9]	77.8 [63.7-82.1]	565.4 [207.2-1542.5]	0.18 [0.06-0.49]	1,082 (1.0)
Tuberculosis	16	87.5 [71.9-88.5]	99.9 [99.8-99.9]	87.5 [71.9-88.5]	1201.8 [297.0-4862.5]	0.13 [0.03-0.46]	306 (0.3)
Nutritional deficiencies	16	68.8 [54.6-75.7]	99.5 [99.2-99.5]	42.3 [34.2-52.4]	125.9 [68.9-230.1]	0.31 [0.15-0.65]	1,226 (1.1)
Hemoglobinopathies and hemolytic anemias	16	62.5 [49.1-71.0]	99.9 [99.7-99.9]	71.4 [55.9-77.8]	429.2 [150.2-1226.9]	0.38 [0.20-0.71]	360 (0.3)
Malaria	14	100.0 [78.5-100.0]	100.0 [99.9-100.0]	93.3 [68.1-99.8]	2749.0 [387.4-19508.4]	-	442 (0.4)
Gynecological diseases	11	81.8 [62.7-84.4]	99.6 [99.4-99.7]	47.4 [37.4-58.3]	225.2 [114.2-443.8]	0.18 [0.05-0.64]	1,536 (1.4)
Neonatal disorders	10	40.0 [29.5-56.0]	99.7 [99.6-99.8]	36.4 [27.3-52.5]	157.3 [54.5-454.1]	0.60 [0.36-1.00]	718 (0.7)
Oral disorders	8	37.5 [27.3-55.8]	99.9 [99.7-99.9]	42.9 [30.3-60.5]	258.3 [68.6-973.0]	0.63 [0.37-1.07]	576 (0.5)
Neglected tropical diseases excluding malaria	7	85.7 [42.1-99.6]	100.0 [99.9-100.0]	100.0 [61.0-100.0]	-	0.14 [0.02-0.88]	361 (0.3)
Leprosy	2	100.0 [15.8-100.0]	100.0 [99.9-100.0]	66.7 [38.7-76.0]	2761.0 [389.1-19593.6]	-	74 (0.1)
Sexually transmitted diseases excluding HIV	1	0.0 [0.0-97.5]	99.8 [99.7-99.8]	0.0 [0.0-43.4]	-	-	187 (0.2)
Sudden infant death syndrome	0	-	100.0 [99.9-100.0]	-	-	-	5 (0.0)
No GBD category	407	53.1 [50.6-55.5]	92.9 [92.3-93.4]	56.4 [53.8-58.9]	7.5 [6.3-8.9]	0.51 [0.46-0.56]	22,450 (20.5)

*Sen* Sensitivity, *Spe* specificity, *PV+* positive predictive value, *LR+* positive likelihood ratio, *LR-* negative likelihood ratio. The version of the classifier used was: using the Word Server Disambiguation server, the expert-based enrichment, and giving priority to the health condition field

### Process of classification of trials

We describe how the classifier performed on the external test set (see Additional file 1 for the process of classification according to the 171 GBD categories).

#### Pathways from trial records to candidate GBD categories

MetaMap annotated 2,600/2,763 (94.1 %) of the trials with at least one UMLS concept. The median (Q1, Q3) number of UMLS concepts per trial was 3 (3, 5) when using the WSD server and 4 (3, 6) without the WSD server. The annotation of all trials involved 2,180 different UMLS concepts. IntraMap projected 1,995/2,180 (91.5 %) UMLS concepts. The median (Q1, Q3) number of ICD10 codes per UMLS concept was 2 (1, 2). The UMLS concepts were projected to 1,361 different ICD10 codes and 1,034/1,361 (76.0 %) ICD10 codes were projected to at least one GBD category.

At this stage, 573/2,180 (26.3 %) UMLS concepts could not be projected to a GBD category. The expert-based enrichment allowed for projecting an additional 41/573 (7.2 %) UMLS concepts.

#### GBD classification

Depending on the version of the classifier, between 594 (21.5 %) and 648 trials (23.5 %) had several candidate GBD categories. With the rule giving priority to the health condition field, the number of trials actually classified with several GBD categories ranged from 177 (6.4 %) to 184 (6.7 %). Without the rule of giving priority to the health condition field, this number ranged from 244 (8.8 %) to 253 (9.2 %). Across all versions of the classifier, the number of trials without GBD classification ranged from 377 (13.6 %) to 414 (15.0 %).

### Evaluation of the classifier

#### Overall performance

The performance of the 8 versions of the classifier is shown in Table 3. The exact-matching proportion was similar for all versions of the classifier. However, the best performance was achieved by using the WSD server, expert-based enrichment, and giving priority to the health condition field (77.8 % of exact-matching). The exact-matching proportion was larger for trials concerning a unique GBD category (82.7 %) and lowest for trials concerning two or more GBD categories (28.6 %). The best version of the classifier was the same for the 171 GBD categories (Additional file 1: Table S3). The performance varied across data sources; overall exact-matching ranged from 66.7 % to 82.2 % (Table 4). When classifying trial records without using the UMLS knowledge source but only using disease names defining the GBD categories, the proportion of clinical trial records from the test set correctly classified to GBD categories was of 51.8 % (Table 3). The knowledge-based classifier had sensitivity and specificity 29.6 % and 5.4 % higher as compared to the baseline not using the UMLS knowledge source.

**Table 3:** Performance of the 8 versions of the classifier, compared to the baseline

	Word Sense Disambiguation	Expert-based enrichment	Priority to health condition field	Exact matching proportion				Weighted average across 28 GBD categories	
				All trials *N* = 2,763	One GBD category *N* = 2,328	Two or more GBD categories *N* = 28	No GBD category *N* = 407	Sensitivity	Specificity
1	Yes	Yes	Yes	77.8	82.7	28.6	53.1	81.9	97.4
2	Yes	Yes	No	77.5	82.5	28.6	52.1	81.8	97.4
3	Yes	No	Yes	76.9	81.4	28.6	54.8	81.0	97.2
4	Yes	No	No	76.9	81.5	28.6	53.8	81.1	97.2
5	No	Yes	Yes	75.6	80.1	28.6	53.1	81.9	97.0
6	No	Yes	No	75.3	79.9	28.6	52.1	81.8	97.0
7	No	No	Yes	74.8	79.0	25.0	54.8	81.0	96.9
8	No	No	No	74.8	79.1	25.0	53.8	81.2	96.9
Baselines		Condition field		48.7	40.5	10.7	98.5	49.3	91.4
		Public title		38.1	27.6	7.1	100.0	38.2	89.6
		Official title		38.0	27.6	7.1	99.3	38.2	89.6
		Three text fields		51.4	43.7	17.9	97.8	52.3	92.0

Exact-matching and weighted averaged sensitivities and specificities for 8 versions of the classifier for the 28 GBD categories, compared to the baseline. Exact-matching corresponds to the proportion (in %) of trials for which the automatic GBD classification is correct. Exact-matching was estimated over all trials (*N* = 2,763), trials concerning a unique GBD category (*N* = 2,328), trials concerning 2 or more GBD categories (*N* = 28), and trials not relevant for the GBD (*N* = 407). The weighted averaged sensitivity and specificity corresponds to the weighted average across GBD categories of the sensitivities and specificities for each GBD category plus the “No GBD” category (in %). The 8 versions correspond to the combinations of the use or not of the Word Sense Disambiguation server during the text annotation, the expert-based enrichment database, and the priority to the health condition field as a prioritization rule. The baseline did not used the UMLS knowledge source, but a clinical trial record was classified to a GBD category if at least one of the disease names defining that GBD category appeared verbatim in the condition field, the public or scientific titles, separately, or in at least one of these three text fields

**Table 4:** Performance of the classifier per source of data for the 28 GBD categories

	Exact-matching (% n/N)				Weighted average across 28 GBD categories	
Source	All trials	One GBD category	No GBD category	Two or more GBD categories	Sensitivity	Specificity
Emdin 2015	66.7 (346/519)	66.4 (300/452)	68.2 (45/66)	100.0 (1/1)	71.5	96.4
Viergever 2013	82.2 (1045/1271)	85.3 (925/1085)	64.5 (120/186)	0.0 (0/0)	86.6	97.8
On going work	77.9 (758/973)	88.5 (700/791)	32.9 (51/155)	25.9 (7/27)	81.3	97.2

Exact-matching and weighted averaged sensitivities and specificities for the classifier to the 28 GBD categories for each source of data. The version of the classifier used was: using the Word Sense Disambiguation server, the expert-based enrichment database and the priority to the health condition field. Exact-matching corresponds to the proportion (in %) of trials for which the automatic GBD classification is correct. Exact-matching was estimated over all trials, trials concerning a unique GBD category, trials concerning 2 or more GBD categories, and trials not relevant for the GBD. The weighted averaged sensitivity and specificity corresponds to the weighted average across GBD categories of the sensitivities and specificities for each GBD category plus the “No GBD” category (in %)

#### Performance for each GBD category

The performance of the best-performing classifier to identify the “Neoplasms” category was excellent (Table 2). The positive likelihood ratio was 38.2 [28.7–50.8] and negative likelihood ratio 0.03 [0.02–0.04]; we can be confident that trials classified as studying “Neoplasms” actually concerned that GBD category, and conversely those not classified as studying “Neoplasms” did not concern the category.

The performance of the classifier in identifying the “Diabetes, urinary diseases and male infertility” and “Cardiovascular and circulatory diseases” categories was good. The specificity of these categories was very high, so a mapping of these categories based on the classifier will not overestimate the effort of research in these fields. However, the sensitivity for these categories was 81.0 % [78.0–83.0] and 75.7 % [72.5–78.1], respectively, so a mapping of these categories may underestimate the effort of research in these fields.

The performance of the classifier in identifying the “Mental and behavioral disorders”, “Musculoskeletal disorders”, “HIV/AIDS” and “Neurological disorders” categories was high. These categories also had high positive likelihood ratios and low negative likelihood ratios. However, the numbers of trials concerning these categories were lower. We cannot conclude on the performance in identifying the remaining GBD categories because of the very low numbers of trials in the external test set (<90 trials per category).

The lowest performance was for the “Injuries” and “Maternal disorders” categories. The “Injuries” category was studied by 56 clinical trials and the sensitivity was low (16.1 % [13.4–23.1]), so a high proportion of trials concerning injuries may not be detected by the classifier. Similarly, the sensitivity for “Maternal disorders” was 39.5 % [33.2–47.6], so the classifier may not detect correctly these trials.

Overall, our classifier identified 407 trials not concerning any GBD category. The sensitivity was low (53.1 % [50.6–55.5]), so half of the trials not concerning any relevant GBD category were actually classified by using GBD categories. The positive predictive value was also low (56.4 % [53.8–58.9]), so half of trials classified as “No GBD” category actually concerned a relevant GBD category.

When classifying trial records without using the UMLS knowledge source but only using disease names defining the GBD categories, the sensitivities were extremely low as compared to those of the knowledge-based classifier for all GBD categories but for semantically simple GBD categories: “HIV/AIDS”, “Hepatitis”, “Tuberculosis”, “Malaria” and “Leprosy” (Additional file 1: Table S4).

Across the 171 GBD categories, the performance was appropriate for the GBD categories most represented in the test set. However, for a high proportion of GBD categories, the number of trials in the test set was not sufficient to conclude on the performance of the classifier in identifying them (Additional file 1: Table S2).

### Classification of all trials registered at the WHO ICTRP

In total, 109,603 interventional trials were classified by using the best-performing version of the classifier (Additional file 2). The number of trials per GBD category is shown in Table 2. The “Neoplasms” category was the most used for classifying clinical trials (22.8 %), followed by “Diabetes, urinary diseases and male infertility” (8.9 %) and “Cardiovascular and circulatory diseases” (8.1 %). In total, 20.5 % of trials could not be classified by a relevant GBD category.

## Discussion

We developed a knowledge-based classifier to automatically map clinical trial records to a 28- and 171-class grouping of the taxonomy of diseases and injuries from the GBD 2010 study. In a validation study, the performance of the classifier was very good for trials of major groups of diseases, including cancer, diabetes and cardiovascular diseases. Our classifier allowed for classifying all trials registered at the WHO ICTRP.

### Comparison to related work

Several studies have previously evaluated the gap between health research and health needs [35, 36, 38–43]. However, in these studies, the classification of health R&D activities was always conducted manually. Manual classification inherently restricted those studies to limited sample sizes, specific medical areas, regions or types of studies. In addition, these studies were not updated. Our automatic classifier can allow for large-scale mapping of all clinical trials registered at the WHO ICTRP (more than 300,000 trials) about all diseases and all regions and the evolution over time.

Previous work used NLP methods to conduct curation of the eligibility criteria field from clinical trial records to improve the retrieval of relevant clinical trials for patients [14–26] In contrast to previous work, we conducted NLP analyses of the condition field and the public and scientific titles from clinical trial records to achieve a different objective, the classification of the condition studied in clinical trials according to a standardized taxonomy of diseases and injuries. Previous studies of automatic indexing used health topics in medical research. The Medical Text Indexer (MTI), developed at the NLM, is used for providing indexing recommendations for data sources such as MEDLINE, PubMed and ClinicalTrials.gov. [29, 44] MTI produces Medical Subject Headings (MeSH) recommendations by combining a statistical method and a natural language processing method based on MetaMap and the Restrict-to-MeSH implemented in IntraMap. This algorithm was shown to be successful for automatically assigning ICD9 codes to radiology reports [45]. To our knowledge no previous work has used the knowledge-based sequence MetaMap - IntraMap to assign GBD categories to clinical trials. The Aggregate Analysis of ClinicalTrials.gov project used indexing with MeSH terms to group trials by medical specialty [30]. However, the medical specialties cannot be connected to the burden of disease. Evans et al. projected all articles indexed in MEDLINE to GBD categories based on indexing publications with MeSH terms from the MTI [46]. The authors linked MeSH terms to ICD9 codes by using the UMLS database. In our work, we directly targeted a classification of texts from trial records by using ICD10 codes because GBD categories are defined with that terminology. Instead of using MeSH terms as an intermediate for projection, which may increase the error rate, we chose to develop our method for classifying automatically health topics according to GBD categories based on ICD10. In addition, we mapped ICD10 codes to GBD categories because the GBD 2010 study provides a burden estimate for each GBD category, and not for each ICD10 code. Moreover, these previous studies focused on the curation of health topics of clinical trials records registered at ClinicalTrials.gov, thereby excluding 31.2 % of trials in the WHO ICTRP [9]. Our method of classification was based on the processing of the condition field and public and scientific titles only, which are required by the WHO ICTRP [47]. Thus, our method can be transposed to any of the 16 clinical trial repositories included in the WHO ICTRP up to date, including clinicaltrials.gov. All these sources of registries are fundamental to conduct a worldwide mapping of registered clinical trials to be compared to global health needs. In addition, in our github repository we include codes to analyze clinical trial records downloaded from WHO ICTRP and clinicaltrials.gov websites.

### Strength of the knowledge-based classifier

Our classifier has several strengths. First, it allows for developing a reliable region-specific mapping of trials, especially in fields such as cancer. Such a mapping can be compared to the region-specific burden of the corresponding diseases. Considering that the classification is imperfect, a region-specific mapping of research topics other than cancer with the classifier should take into account the possible misclassification. Second, the classifier of clinical trials we developed may be used for conducting semi- and fully-automatic classification recommendations. Machine learning methods based on the characteristics of trial records and on the pathways drawn between trials and GBD categories may allow for identifying trials for which the classifier does not show a confident classification. These trials may be considered for manual revision. Because the WHO ICTRP database is large and constantly growing, manual revisions may be expensive. Crowd-sourcing based on the interface for the manual classification we developed could be scaled up to divide the effort needed for revision. In addition, trial registries such as ClinicalTrials.gov could include the GBD classification as a mandatory field in trial records. The classifier we developed could provide an automatic recommendation for classification of newly registered trials by the GBD categories, thus reducing the burden of registration. Another strength of the classifier is that it is based on the UMLS Knowledge Source, a metathesaurus widely used for analyzing biomedical text, which increases the portability and reproducibility of the classification. The classification method development did not rely on data in the test set. Other approaches such as statistical methods of classification (e.g. support vector machines) may be used to address our objective. However, our knowledge-based classifier may be more resilient to the evolution of clinical trial records. Every year, about 20,000 new clinical trials are registered at WHO ICTRP [9]. Statistical methods of classification would need new training data to perform classification out of the rule space of a training dataset. Another strength is that our knowledge-based classifier allows understanding the process of classification of trial records (Fig. 1), as compared to statistical classifiers. For a public health project, it is of great value understanding the process of data curation [48, 49]. In addition, the approach is generalizable to other sources such as grants, articles, and systematic reviews.

### Performance of the knowledge-based classifier

The evaluation of our classifier on a gold standard external test set yielded an overall performance of 81.9 % sensitivity and 97.6 % specificity. Overall, 77.8 % of trial records from the external test set were correctly classified towards a 28-class grouping of the GBD cause list. Pradhan et al. evaluated the performance of 17 systems to normalize disorder mentions in biomedical text using a standardized ontology, the Systematized Nomenclature of Medicine—Clinical Terms (SNOMED CT) [50, 51]. In that study, the best performing system correctly normalized 58.9 % of disorder mentions. It is hard to compare this performance to the performance of our classifier, as the input space (biomedical text vs clinical trial records) and the target spaces (SNOMED CT vs GBD categories) differ. However, we consider that the performance of the classifier was satisfactory for trials concerning majors groups of diseases as cancer, diabetes and cardiovascular diseases. In particular, we can be confident on the mapping provided by the classifier of clinical trials concerning cancer. In addition, the classifier may not overestimate the effort of research in diabetes and cardiovascular diseases. Our classifier performed differently across data sources. This may be explained because the different these data sources can not be considered as random samples of clinical trials. However, we could identify some GBD categories for which the overall performance of the classifier was excellent.

### Limitations

Our work has several limitations. First, the quality of the mapping of health research depends on the quality of the registration of clinical trials. Trial registration remains of low quality, but endorsements from WHO are attempting to improve the registration system [7, 47]. In addition, the misclassification of diseases may be correlated to trial location. For instance, our classifier only supports English language, as MetaMap identifies UMLS concepts in biomedical text written in English. This may increase the misclassification in non-English speaking countries. However, according to the International Standards for Clinical Trial Registries from the WHO, all items of trial records included in the WHO ICTRP (including the condition field and the public and scientific titles) must be available in English language [47]. Similarly, compliance to registration of clinical trials may vary across regions. However, it is unlikely that compliance on registration vary across diseases. Therefore, in regions with low compliance of registration, a lower number of clinical trials concerning a disease as compared to other diseases may effectively correspond to a gap of health research. Second, our classifier may poorly identify some categories. For instance, the sensitivity for the “Injuries” category, accounting for 10.7 % of the global burden in 2010, was low [27]. In our test set, clinical trials concerning injuries mainly studied the adverse effects of medical treatments (35/56). In these trials, the classifier is more likely to identify the health condition targeted by those medical treatments rather than considering that the clinical trials studied the adverse effects of the treatments. Thus, this misclassification may not be considered an error in the mapping because trials studying the adverse effects of the treatment used for a certain condition will be conducted in countries where that particular condition is a burden. Third, the classifier may poorly identify trials not concerning any relevant GBD category. For the classifier to identify a “No GBD” category trial, it needs to be unable to project the trial to any GBD category. However, any UMLS concept recognized in the trial record projected to a GBD category will lead to a classification of the trial. The suppression of noise candidate GBD categories by using the prioritization rules do not allow for suppressing all the candidate GBD categories but rather only choosing the most accurate classification among the candidates. However, the specificities of each of the 28 GBD categories were generally high, so the number of “No GBD” category trials wrongly classified remained low per GBD category.

In our 28-class grouping of diseases and injuries we excluded two residual categories from the GBD cause list, “Other infectious diseases” and “Other endocrine, nutritional, blood, and immune disorders”, accounting for 1.2 % of the global burden in 2010. These residual categories are difficult to cover as they are defined using sets of ICD10 to complement the major diseases groups, and are thus particularly large and complex. We decided no to take into account these categories because these coverings may add much complexity to the classification tasks with very small benefits in terms of global mapping of clinical research. Actually, we considered that these categories would not be informative for the purposes of developing a global mapping of registered clinical trials across diseases to be compared to health needs. Finally, in our study, we considered the particular taxonomy of the US Institute for Health Metrics and Evaluation for the GBD 2010 Study. This taxonomy may not be perfectly suitable for conducting a mapping of health R&D. For instance, health conditions that may be considered public health priorities in some regions, such as obesity, venous thromboembolism or heart failure, are part of the residual categories. However, the GBD study is a worldwide effort to estimate the evolution of the burden of all diseases in all countries in the world. It provides a consensual taxonomy of diseases for use in comparing the research effort to the burden of diseases.

## Conclusion

Herein, we presented a knowledge-based classifier to map the health conditions studied in registered clinical trials according to the taxonomy of diseases and injuries from the Global Burden of Diseases 2010 study. The overall performance of the classifier was 81.9 % sensitivity and 97.6 % specificity. We applied it to the entire WHO ICTRP database, which characterizes the global burden of disease addressed by the 109,603 clinical trials in the database. This classifier allows for comparing the research effort to the disease burden on a large scale for all diseases and all regions and studying the evolution over time.

## Additional files

Additional file 1: Includes details on the implementation of MetaMap and IntraMap, prioritization rules, the test set of clinical trials and the classification of the external test set according to the 171 GBD categories. **Dataset S1:** Expert-based enrichment database for the classification according to the 28 GBD categories. Manual classification of 503 UMLS concepts that could not be mapped to any of the 28 GBD categories. **Dataset S2:** Expert-based enrichment database for the classification according to the 171 GBD categories. Manual classification of 655 UMLS concepts that could not be mapped to any of the 171 GBD categories, among which 108 could be projected to candidate GBD categories. **Table S1:** Excluded residual GBD categories for the grouping of the GBD cause list in 171 GBD categories. A grouping of 193 GBD categories was defined during the GBD 2010 study to inform policy makers about the main health problems per country. From these 193 GBD categories, we excluded the 22 residual categories listed in the Table. We developed a classifier for the remaining 171 GBD categories. Among these residual categories, the unique excluded categories in the grouping of 28 GBD categories were “Other infectious diseases” and “Other endocrine, nutritional, blood, and immune disorders”. **Table S2:** Per-category evaluation of performance of the classifier for the 171 GBD categories plus the “No GBD” category. Number of trials per GBD category from the test set of 2,763 clinical trials. Sensitivities, specificities (in %) and likelihood ratios for each of the 171 GBD categories plus the “No GBD” category for the classifier using the Word Sense Disambiguation server, the expert-based enrichment database and the priority to the health condition field. **Table S3:** Performance of the 8 versions of the classifier for the 171 GBD categories. Exact-matching and weighted averaged sensitivities and specificities for 8 versions of the classifier for the 171 GBD categories. Exact-matching corresponds to the proportion (in %) of trials for which the automatic GBD classification is correct. Exact-matching was estimated over all trials (*N* = 2,763), trials concerning a unique GBD category (*N* = 2,092), trials concerning 2 or more GBD categories (*N* = 187), and trials not relevant for the GBD (*N* = 484). The weighted averaged sensitivity and specificity corresponds to the weighted average across GBD categories of the sensitivities and specificities for each GBD category plus the “No GBD” category (in %). The 8 versions correspond to the combinations of the use or not of the Word Sense Disambiguation server during the text annotation, the expert-based enrichment database, and the priority to the health condition field as a prioritization rule. **Table S4:** Per-category evaluation of the performance of the baseline for the 28 GBD categories plus the “No GBD” category. Number of trials per GBD category from the test set of 2,763 clinical trials. Sensitivities and specificities (in %) of the 28 GBD categories plus the “No GBD” category for the classification of clinical trial records towards GBD categories without using the UMLS knowledge source but based on the recognition in free text of the names of diseases defining in each GBD category only. For the baseline a clinical trial records was classified with a GBD category if at least one of the 291 disease names from the GBD cause list defining that GBD category appeared verbatim in the condition field, the public or scientific titles, separately, or in at least one of these three text fields. (DOCX 84 kb) Additional file 2: Classification towards the 28-class grouping of GBD categories of all interventional trials registered at WHO ICTRP before February 2014. Classification of *N* = 109,603 clinical trials using the best-performing version of the classifier (using the Word Sense Disambiguation server, the expert-based enrichment database and the priority to the health condition field. (XLSX 12447 kb)

## Abbreviations

GBD: Global Burden of Diseases
ICD10: International classification of diseases 10th version
ICTRP: International clinical trials registry platform
LR+/-: Positive and negative likelihood ratios
MeSH: Medical subject headings
MTI: Medical text indexer
NLM: National library of medicine
NLP: Natural language processing
R&D: Research and development
SNOMED CT: Systematized nomenclature of medicine—clinical terms
UMLS: Unified medical language system
WHO: World Health Organization
WSD: Word sense disambiguation

## Footnotes

1 Body Mass Index
