# Supplementary material for: Automatic classification of registered clinical trials towards the Global Burden of Diseases taxonomy of diseases and injuries
Source: BMC Bioinformatics. 2016 Sep 22;17:392. doi: 10.1186/s12859-016-1247-7 (PMC5034670; doi:10.1186/s12859-016-1247-7)
Supplement: Additional file 1: — Includes details on the implementation of MetaMap and IntraMap, prioritization rules, the test set of clinical trials and the classification of the external test set according to the 171 GBD categories. Dataset S1: Expert-based enrichment database for the classification according to the 28 GBD categories. Manual classification of 503 UMLS concepts that could not be mapped to any of the 28 GBD categories. Dataset S2: Expert-based enrichment database for the classification according to the 171 GBD categories. Manual classification of 655 UMLS concepts that could not be mapped to any of the 171 GBD categories, among which 108 could be projected to candidate GBD categories. Table S1: Excluded residual GBD categories for the grouping of the GBD cause list in 171 GBD categories. A grouping of 193 GBD categories was defined during the GBD 2010 study to inform policy makers about the main health problems per country. From these 193 GBD categories, we excluded the 22 residual categories listed in the Table. We developed a classifier for the remaining 171 GBD categories. Among these residual categories, the unique excluded categories in the grouping of 28 GBD categories were “Other infectious diseases” and “Other endocrine, nutritional, blood, and immune disorders”. Table S2: Per-category evaluation of performance of the classifier for the 171 GBD categories plus the “No GBD” category. Number of trials per GBD category from the test set of 2,763 clinical trials. Sensitivities, specificities (in %) and likelihood ratios for each of the 171 GBD categories plus the “No GBD” category for the classifier using the Word Sense Disambiguation server, the expert-based enrichment database and the priority to the health condition field. Table S3: Performance of the 8 versions of the classifier for the 171 GBD categories. Exact-matching and weighted averaged sensitivities and specificities for 8 versions of the classifier for the 171 GBD categories. Exact-matching corresponds to the propo [file 12859_2016_1247_MOESM1_ESM.docx]

**Automatic classification of registered clinical trials towards the Global Burden of Diseases taxonomy of diseases and injuries**

**Supplementary Information**

**APPENDIX S1: Supplementary information for methods**

**MetaMap implementation**

We used the MetaMap version metamap14, the lexicon version used was 2014 and the database used was USAbase 2014AB.

We restricted the output of MetaMap to concepts included in the semantic group DISORDERS, with the exception of concepts of semantic type “Findings”. We considered the following semantic types:

- Acquired Abnormality
- Anatomical Abnormality
- Cell or Molecular Dysfunction
- Congenital Abnormality
- Disease or Syndrome
- Experimental Model of Disease
- Injury or Poisoning
- Mental or Behavioral Dysfunction
- Neoplastic Process
- Pathologic Function
- Sign or Symptom

We used the strict-model parametrization of MetaMap.

We developed the classifier using and not the Word Sense Disambiguation server.

The metamap options used were: -J acab,anab,comd,cgab,dsyn,emod,inpo,mobd,neop,patf,sosy -AvN -V USAbase (-y)

For a high proportion of clinical trial records, the health condition field corresponds to a list of diseases. We indexed separately each item of the list. For the public title and scientific title, the entire text was indexed.

**IntraMap implementation**

We used the IntraMap version 2014 based on the USAbase 2014AB.

For each UMLS concept, the IntraMap output is a list of ICD10 codes. This list can be found by four different means: by synonymy, by built-in relations, through the graph of ancestors and based on other mappings. Mappings through synonymy are expected to have the highest quality, followed by mappings through built-in relations. Follows mappings through the graph of ancestors, which generally mapped to a more general concept. We excluded mappings using other means than the three aforementioned.

In some cases, IntraMap's output is empty. For instance, concepts C0012634 “Disease”, or C0009566 “Complication”, could not be projected by IntraMap to ICD10 codes.

**Punctual reparations to MetaMap and IntraMap**

During the development of the classifier, we identified several errors of MetaMap or IntraMap leading to misclassification. These reparations are explained bellow:

- IntraMap projected the UMLS concept C1306459, “Primary malignant neoplasms”, to the ICD10 code C72.9, “Malignant neoplasm of central nervous system, unspecified”. We changed this projection to C00-C97, “Malignant neoplasms”.
- IntraMap projected the UMLS concept C0876994, “Cardiotoxicity” to the ICD10 code M10.2, “Drug-induced gout”. We suppressed this projection and leaved the concept without projection.
- IntraMap projected the UMLS concept C2825032, “Withdrawal (dysfunction)” to the ICD10 code F91.2 “Socialized conduct disorder”, leading to the GBD category “Mental and behavioral disorders”. However, the annotation from MetaMap using the “Withdrawal (dysfunction)” UMLS concept arrived when recognizing in text the term “withdrawal”, generally corresponding to a specification of the design of the study rather than the health condition studied. We suppressed this projection and leaved the concept without projection.
- The UMLS concept C0949179, “Edentulism” could not be projected to any ICD10 code. However, the GBD study reserves a special GBD category to that health condition. We projected that ICD10 code directly to the “Oral disorders” category.
- IntraMap projected the UMLS concept C0878773, “Overactive Bladder” to several ICD10 codes, among which G00-G99.9, “Diseases of the nervous system”. We suppressed the projection to that ICD10 code.
- IntraMap projected the UMLS concept C0242656, “Disease Progression” to the ICD10 code C00-D48.9, “Neoplasms”. We suppressed this projection and leaved the concept without projection.
- IntraMap projected the UMLS concept C0687702, “Cancer Remission” to the ICD10 code C00-D48.9, “Neoplasms”. However, the annotation from MetaMap using the “Cancer Remission” UMLS concept arrived when recognizing in text the term “remission”, which could correspond to the remission of any disease other than cancer. We suppressed this projection and leaved the concept without projection.

**Prioritization rules**

Prioritization rules corresponded to a set of algorithms to derive the GBD classification of a trial based on the pathways issued from the trial record to candidate GBD categories. Pathways from the trial record to candidate GBD categories were derived from successive projections: from the trial record to free text, from free text to UMLS concepts, from UMLS concepts to ICD10 codes, and from ICD10 codes to GBD categories. The rules of prioritization gave priority to candidate GBD categories consistently achieved by the pathways, as compared to candidate GBD categories achieved by isolated pathways. At each projection, noise GBD categories may appear. For instance, noise candidate GBD categories may appear when annotating the scientific title of the trial using a UMLS concept revealing of the design of the study rather than the health condition studied. Similarly, the UMLS concept “Breast neoplasms” may be projected to both ICD10 codes “Malignant neoplasms” and “Malignant breast neoplasms”, leading to an UMLS concept having as candidate GBD categories all the 27/171 GBD categories corresponding to cancers.

Prioritization rules were then used at different stages of the projections. First, to deriving a projection of each UMLS concept to GBD categories without noise, based on the projection to GBD categories of the ICD10 codes corresponding to that UMLS concept. Second, to deriving a projection of each text field (health condition, public title and scientific title) without noise based on the projection to GBD categories of the UMLS concepts used for annotating each text. Third and finally, to deriving a projection of the trial record based on the projection to GBD categories of each of its text fields.

The first two utilizations of the prioritization rules used the same algorithm described bellow. The final prioritization rule used an algorithm giving particular priority to the projection to GBD categories of the health condition field as compared to the public and scientific titles.

The algorithm used for the first two stages of projection was based on gaving priority to smaller lists and to intersections of GBD categories between lists. Given a set *E* of lists of GBD categories (e.g. the list of candidate GBD categories per UMLS concept annotating a given text field), the algorithm derived a final list *L* of GBD categories (e.g. corresponding to the GBD classification of the text field) by adding progressively to *L* eligible GBD categories. We initialized the list of GBD categories *L* with all the elements of *E* of size *k*=1. Then, for *k*>1 in increasing order:

1. We considered *E_k* as the set of lists of *E* of size *k*.
2. We suppressed from *E_k* all the lists having a non-empty intersection with *L*.
3. We considered *I_k* as the global intersection of all the lists of *E_k*. If *I_k* was non empty, we added *I_k* to *L*. If *I_k* was empty, we added to *L* all the elements included in the lists of *E_k*.

The algorithm used for the final prioritization rule to derive the GBD classification of the trial record based on the projection to GBD categories of the corresponding text fields was as follows:

1. If the condition field had a unique candidate GBD category, we considered it as the GBD classification of the trial.
2. If the condition field had multiple candidate GBD categories, we tested if the intersection of these categories with those appearing in the other text fields was non-empty. If it was non-empty, the GBD classification of the trail was the aforementioned intersection. If it was empty, the GBD classification of the trial was the set of candidate GBD categories of the condition field.
3. If the condition field did not have any candidate GBD category, we derived the GBD classification by considering the candidate GBD categories from the public title and the scientific title fields. We gave priority to the GBD categories appearing in both text fields, and if the intersection was empty, to the union.

When we suppressed the priority to the condition field from the classifier, the final GBD classification of the trial was derived from GBD classifications of each text field using the same algorithm as in the first two prioritization rules.

**Test set of clinical trials**

For trials classified in more than one data source, we gave priority to the classification from the ESORT study, then from *Viergever et al*, and finally from the ongoing study from our team.

From the ESORT study, we had a manual classification of 519 unique trials having valid registration number and included in ICTRP. In the study from *Viergever et al.,* trial records were classified using the GBD categories as defined in the GBD study conducted by the World Health Organization (WHO) (Table C3 in *The Global Burden of Disease: 2004 update (2008))*. The GBD categories defined by the WHO may differ with the GBD cause list from the GBD 2010 study conducted by the Institute for Health Metrics and Evaluation (IHME). In the GBD study from the WHO, GBD categories are also defined using ICD10 codes. We identified the GBD categories from the WHO for which all ICD10 codes were included in a unique GBD category among the 171 groups of GBD categories we defined from the GBD cause list of the IHME. We excluded all trials classified using a GBD category from the WHO for which the ICD10 codes could be projected to two or more GBD categories. For instance, the GBD category from the WHO “Melanoma and other skin cancers” (C43-C44) could correspond to the GBD categories from the IHME “Malignant melanoma of skin” (C43,D03,D48.5) or “Non-melanoma skin cancer” (C44,D04). From that sample, we did not exclude trials classified as “No GBD” category, as we considered they corresponded to trials without GBD category for both taxonomies, WHO and IHME. We excluded 1,105 trials. We also excluded 5 trials already classified during the ESORT study. From the manual classification of the researcher of our team, we had 1,001 trials. In total, 28 trials were classified in the other data sources and were excluded. In total, we disposed of 2,381 unique clinical trials.

During the ESORT study, trials were classified using the 193 categories from the GBD cause list. In the study from *Viergever et al.* trials were reclassified to the 171 GBD categories defined in our study using the aforementioned rule. The physician from our team classified trials directly using the 171 GBD categories defined in our study.

For each trial we identified the GBD categories corresponding to the classification among the 28 and 171 GBD categories. For instance, when a trial from the ESORT study was classified using a residual category excluded from the 28 or 171 GBD categories, we classified it as “No GBD” category.

**APPENDIX S2: Classification of the external validation dataset according to the 171 GBD categories**

Across 2,763 trial records, 2,092 (75.7%) concerned a unique GBD category, 187 (6.8%) concerned two or more GBD categories, and 484 (17.5%) concerned health conditions from the residual categories that we excluded or health conditions not relevant for the GBD 2010 study. A majority of clinical trials studied the “Breast cancer” category (232 trials), followed by “Leukemia” (225 trials) and “Diabetes mellitus” (202 trials) (Table S2). In our test set, trials concerned 128/171 GBD categories.

**Process of classification of trials**

The stages of text annotation and projection of UMLS concepts to ICD10 codes are identical than for the classification to 28 GBD categories.

In total, 623/1361 (45.8%) ICD10 codes were projected to at least one GBD category. The median (Q1, Q3) number of GBD categories per projected ICD10 code was 1 (1, 1).

At this stage, 965/2180 (44.2%) UMLS concepts could not be projected to a GBD category. In the expert-based enrichment database we found manual revision for 403/965 (41.8%) not projected UMLS concepts, over which 68 were manually projected to a GBD category.

**Evaluation of the classifier**

*Overall performances*

The performances of classification of the 2,763 trial records for the eight versions of the classifier are shown in Table S3. The unique option of the versions of the classifier that substantially improved the performances of classification was the use of the expert-based enrichment database (between 6 and 7% of improvement). The best performances (74.0% of exact-matching) were achieved using the WSD server, the expert-based enrichment database and giving priority to the health condition field. The exact-matching was higher for trials concerning a unique GBD category (77.4%) and was the lowest for trials concerning two or more GBD categories (43.3%).

*Performances for each GBD category*

The performances for each GBD category for the version of the classifier using the WSD, the expert-based enrichment and the priority to the condition are shown in Table S2.

For all GBD categories with a sufficient high number of trials, the specificity was consistently high (more than 50 trials). This means that the classifier generally does not underestimate the effort of research for these GBD categories. The 27 types of cancers among the 171 GBD categories have generally a low sensitivity, and are studied by a similar amount of trials. This is because trials in the test set we considered 95 trials were classified as “Neoplasms”, meaning that they were classified using all the 27 types of cancers among the 171 categories. The low sensitivity of these categories may be explained because some of the trials concerning all cancers may be classified using specific cancers.

**SUPPLEMENTARY TABLES**

**Table S1: Excluded residual GBD categories for the grouping of the GBD cause list in 171 GBD categories**

| Excluded GBD categories |
| --- |
| Other neglected tropical diseases |
| Other neonatal disorders |
| Other nutritional deficiencies |
| Other sexually transmitted diseases |
| Other infectious diseases |
| Other neoplasms |
| Other cardiovascular and circulatory diseases |
| Other chronic respiratory diseases |
| Other digestive diseases |
| Other neurological disorders |
| Other mental and behavioral disorders |
| Other urinary diseases |
| Other gynecological diseases |
| Other hemoglobinopathies and hemolytic anemias |
| Other endocrine, nutritional, blood, and immune disorders |
| Other muskuloskeletal disorders |
| Other skin and subcutaneous diseases |
| Other hearing loss |
| Other vision loss |
| Other sense organ diseases |
| Other transport injury |
| Unintentional injuries not classified elsewhere |

A grouping of 193 GBD categories was defined during the GBD 2010 study to inform policy makers on the main health problems per country. From these 193 GBD categories, we excluded the 22 residual categories listed in the Table. We developed a classifier to the remaining 171 GBD categories. Among these residual categories, the unique excluded categories in the grouping of 28 GBD categories were “Other infectious diseases” and “Other endocrine, nutritional, blood, and immune disorders”.

**Table S2: Per-category evaluation of performance of the classifier for the 171 GBD categories plus the “No GBD” category**

| GBD categories | Number of trials | Sensitivity | Specificity | Positive Likelihood Ratio | Negative Likelihood Ratio |
| --- | --- | --- | --- | --- | --- |
| **Tuberculosis** | 16 | 87.5 [71.9-88.5] | 99.9 [99.8-99.9] | 1201.8 [297.0-4862.5] | 0.13 [0.03-0.46] |
| **HIV/AIDS** | 97 | 88.7 [83.9-90.4] | 99.7 [99.5-99.7] | 295.5 [147.4-592.3] | 0.11 [0.07-0.20] |
| **Diarrhea, lower respiratory infections, meningitis, and other common infectious diseases** |  |  |  |  |  |
| Diarrheal diseases | 1 | 100.0 [20.7-100.0] | 99.9 [99.8-99.9] | 1381.0 [345.6-5519.1] | NaN |
| Typhoid and paratyphoid fevers | 0 | NaN | 100.0 [99.9-100.0] | NaN | NaN |
| Lower respiratory infections | 25 | 84.0 [72.3-86.7] | 99.5 [99.3-99.6] | 176.9 [100.2-312.4] | 0.16 [0.07-0.39] |
| Upper respiratory infections | 13 | 53.8 [40.8-65.1] | 99.9 [99.7-99.9] | 493.6 [143.1-1702.1] | 0.46 [0.26-0.83] |
| Otitis media | 1 | 100.0 [20.7-100.0] | 100.0 [99.9-100.0] | NaN | NaN |
| Meningitis | 4 | 100.0 [51.0-100.0] | 99.3 [99.0-99.4] | 138.0 [89.1-213.5] | NaN |
| Encephalitis | 0 | NaN | 99.3 [99.1-99.4] | NaN | NaN |
| Diphtheria | 1 | 100.0 [20.7-100.0] | 99.9 [99.8-99.9] | 1381.0 [345.6-5519.1] | NaN |
| Whooping cough | 0 | NaN | 99.9 [99.8-99.9] | NaN | NaN |
| Tetanus | 2 | 100.0 [34.2-100.0] | 99.9 [99.8-99.9] | 1380.5 [345.4-5517.1] | NaN |
| Measles | 2 | 50.0 [29.3-70.7] | 100.0 [99.9-100.0] | NaN | 0.50 [0.13-2.00] |
| Varicella | 0 | NaN | 100.0 [99.9-100.0] | NaN | NaN |
| **Malaria** | 14 | 100.0 [78.5-100.0] | 100.0 [99.8-99.9] | 2749.0 [387.4-19508.4] | NaN |
| **Neglected tropical diseases excluding malaria** |  |  |  |  |  |
| Chagas disease | 0 | NaN | 100.0 [99.9-100.0] | NaN | NaN |
| Leishmaniasis | 0 | NaN | 100.0 [99.9-100.0] | NaN | NaN |
| African trypanosomiasis | 0 | NaN | 100.0 [99.9-100.0] | NaN | NaN |
| Schistosomiasis | 0 | NaN | 100.0 [99.8-99.9] | NaN | NaN |
| Cysticercosis | 0 | NaN | 100.0 [99.8-99.9] | NaN | NaN |
| Echinococcosis | 0 | NaN | 100.0 [99.8-99.9] | NaN | NaN |
| Lymphatic filariasis | 0 | NaN | 100.0 [99.8-99.9] | NaN | NaN |
| Onchocerciasis | 0 | NaN | 100.0 [99.8-99.9] | NaN | NaN |
| Trachoma | 0 | NaN | 100.0 [99.9-100.0] | NaN | NaN |
| Dengue | 3 | 100.0 [43.9-100.0] | 100.0 [99.9-100.0] | NaN | NaN |
| Yellow fever | 1 | 100.0 [20.7-100.0] | 100.0 [99.9-100.0] | NaN | NaN |
| Rabies | 0 | NaN | 100.0 [99.9-100.0] | NaN | NaN |
| Ascariasis | 1 | 0.0 [0.0-79.3] | 100.0 [99.8-99.9] | NaN | NaN |
| Trichuriasis | 0 | NaN | 100.0 [99.8-99.9] | NaN | NaN |
| Hookworm disease | 1 | 100.0 [20.7-100.0] | 100.0 [99.9-100.0] | NaN | NaN |
| Food-borne trematodiases | 1 | 100.0 [20.7-100.0] | 100.0 [99.8-99.9] | 2762.0 [389.2-19600.7] | NaN |
| **Maternal disorders** | 43 | 39.5 [33.2-47.6] | 99.8 [99.6-99.8] | 179.2 [74.3-432.4] | 0.61 [0.48-0.77] |
| **Neonatal disorders** |  |  |  |  |  |
| Preterm birth complications | 8 | 50.0 [35.5-64.5] | 100.0 [99.8-99.9] | 1377.5 [172.3-11009.9] | 0.50 [0.25-1.00] |
| Neonatal encephalopathy (birth asphyxia and birth trauma) | 1 | 0.0 [0.0-79.3] | 100.0 [99.9-100.0] | NaN | NaN |
| Sepsis and other infectious disorders of the newborn baby | 0 | NaN | 100.0 [99.9-100.0] | NaN | NaN |
| **Nutritional deficiencies** |  |  |  |  |  |
| Protein-energy malnutrition | 3 | 33.3 [24.0-61.3] | 99.8 [99.7-99.8] | 184.0 [29.7-1140.5] | 0.67 [0.30-1.49] |
| Iodine deficiency | 0 | NaN | 100.0 [99.8-99.9] | NaN | NaN |
| Vitamin A deficiency | 0 | NaN | 100.0 [99.9-100.0] | NaN | NaN |
| Iron-deficiency anemia | 9 | 88.9 [66.7-87.8] | 99.6 [99.4-99.7] | 244.8 [126.5-473.8] | 0.11 [0.02-0.71] |
| **Sexually transmitted diseases excluding HIV** |  |  |  |  |  |
| Syphilis | 0 | NaN | 100.0 [99.8-99.9] | NaN | NaN |
| Sexually transmitted chlamydial diseases | 1 | 0.0 [0.0-79.3] | 100.0 [99.8-99.9] | NaN | NaN |
| Gonococcal infection | 0 | NaN | 100.0 [99.8-99.9] | NaN | NaN |
| Trichomoniasis | 0 | NaN | 100.0 [99.8-99.9] | NaN | NaN |
| **Hepatitis** |  |  |  |  |  |
| Acute hepatitis A | 17 | 47.1 [36.7-58.5] | 100.0 [99.8-99.9] | 1292.2 [170.8-9774.8] | 0.53 [0.34-0.83] |
| Acute hepatitis B | 17 | 70.6 [56.6-77.0] | 99.9 [99.7-99.9] | 484.6 [173.7-1352.2] | 0.29 [0.14-0.62] |
| Acute hepatitis C | 17 | 0.0 [0.0-18.4] | 100.0 [99.9-100.0] | NaN | NaN |
| Acute hepatitis E | 17 | 0.0 [0.0-18.4] | 100.0 [99.9-100.0] | NaN | NaN |
| **Leprosy** | 2 | 100.0 [34.2-100.0] | 100.0 [99.8-99.9] | 2761.0 [389.1-19593.6] | NaN |
| **Neoplasms** |  |  |  |  |  |
| Esophageal cancer | 111 | 73.0 [68.0-76.4] | 98.2 [97.9-98.4] | 41.2 [30.3-55.9] | 0.28 [0.20-0.37] |
| Stomach cancer | 135 | 77.8 [73.5-80.6] | 98.6 [98.3-98.8] | 55.2 [39.6-77.0] | 0.23 [0.16-0.31] |
| Liver cancer | 169 | 81.1 [77.4-83.4] | 98.1 [97.8-98.3] | 42.9 [32.2-57.2] | 0.19 [0.14-0.26] |
| Larynx cancer | 103 | 71.8 [66.7-75.4] | 98.2 [97.8-98.4] | 39.0 [28.8-52.8] | 0.29 [0.21-0.39] |
| Trachea, bronchus, and lung cancers | 190 | 83.7 [80.3-85.7] | 98.3 [98.0-98.5] | 50.1 [37.0-67.8] | 0.17 [0.12-0.23] |
| Breast cancer | 232 | 84.9 [82.0-86.7] | 98.2 [97.9-98.4] | 47.8 [35.6-64.1] | 0.15 [0.11-0.21] |
| Cervical cancer | 110 | 71.8 [66.8-75.3] | 98.3 [98.0-98.5] | 42.3 [31.0-57.9] | 0.29 [0.21-0.39] |
| Uterine cancer | 97 | 70.1 [64.8-73.9] | 98.3 [98.0-98.5] | 40.6 [29.7-55.6] | 0.30 [0.22-0.41] |
| Prostate cancer | 151 | 80.1 [76.2-82.6] | 98.3 [98.0-98.5] | 47.6 [35.1-64.4] | 0.20 [0.15-0.28] |
| Colon and rectum cancers | 165 | 80.0 [76.2-82.4] | 98.1 [97.7-98.3] | 41.6 [31.3-55.3] | 0.20 [0.15-0.28] |
| Mouth cancer | 104 | 72.1 [67.0-75.7] | 98.2 [97.8-98.4] | 39.1 [28.9-52.9] | 0.28 [0.21-0.39] |
| Nasopharynx cancer | 99 | 68.7 [63.4-72.6] | 98.3 [97.9-98.5] | 39.8 [29.0-54.6] | 0.32 [0.24-0.43] |
| Cancer of other part of pharynx and oropharynx | 103 | 72.8 [67.7-76.3] | 98.2 [97.9-98.4] | 40.4 [29.8-54.7] | 0.28 [0.20-0.38] |
| Gallbladder and biliary tract cancer | 100 | 70.0 [64.7-73.8] | 98.3 [97.9-98.5] | 40.5 [29.6-55.5] | 0.31 [0.23-0.41] |
| Pancreatic cancer | 127 | 75.6 [71.1-78.6] | 98.2 [97.9-98.4] | 42.4 [31.4-57.2] | 0.25 [0.18-0.34] |
| Malignant melanoma of skin | 104 | 76.0 [70.9-79.2] | 98.1 [97.8-98.3] | 40.4 [30.1-54.3] | 0.24 [0.17-0.34] |
| Non-melanoma skin cancer | 96 | 69.8 [64.4-73.6] | 98.3 [98.0-98.5] | 40.5 [29.5-55.5] | 0.31 [0.23-0.42] |
| Ovarian cancer | 122 | 74.6 [69.9-77.7] | 98.3 [97.9-98.4] | 42.8 [31.6-58.1] | 0.26 [0.19-0.35] |
| Testicular cancer | 96 | 68.8 [63.4-72.7] | 98.3 [98.0-98.5] | 40.7 [29.6-56.1] | 0.32 [0.24-0.43] |
| Kidney and other urinary organ cancers | 133 | 78.9 [74.6-81.6] | 98.3 [98.0-98.5] | 46.1 [34.1-62.4] | 0.21 [0.15-0.30] |
| Bladder cancer | 111 | 73.0 [68.0-76.4] | 98.3 [97.9-98.5] | 42.1 [30.9-57.2] | 0.28 [0.20-0.37] |
| Brain and nervous system cancers | 111 | 82.9 [78.2-85.3] | 98.0 [97.6-98.2] | 40.7 [30.9-53.7] | 0.17 [0.12-0.26] |
| Thyroid cancer | 102 | 69.6 [64.4-73.4] | 98.3 [97.9-98.5] | 40.3 [29.4-55.1] | 0.31 [0.23-0.41] |
| Hodgkin's disease | 98 | 69.4 [64.1-73.2] | 98.0 [97.6-98.2] | 34.2 [25.5-46.0] | 0.31 [0.23-0.42] |
| Non-Hodgkin lymphoma | 145 | 76.6 [72.4-79.4] | 97.6 [97.3-97.9] | 32.3 [24.9-42.0] | 0.24 [0.18-0.32] |
| Multiple myeloma | 138 | 77.5 [73.3-80.3] | 97.9 [97.5-98.1] | 36.3 [27.6-47.8] | 0.23 [0.17-0.31] |
| Leukemia | 225 | 83.6 [80.5-85.5] | 98.1 [97.7-98.3] | 43.3 [32.6-57.5] | 0.17 [0.12-0.23] |
| **Cardiovascular and circulatory diseases** |  |  |  |  |  |
| Rheumatic heart disease | 1 | 0.0 [0.0-79.3] | 99.6 [99.4-99.7] | NaN | NaN |
| Ischemic heart disease | 191 | 77.0 [73.4-79.5] | 99.5 [99.2-99.5] | 141.4 [83.4-239.8] | 0.23 [0.18-0.30] |
| Cerebrovascular disease | 14 | 85.7 [68.9-87.2] | 99.3 [99.1-99.4] | 124.0 [75.5-203.8] | 0.14 [0.04-0.52] |
| Hypertensive heart disease | 2 | 0.0 [0.0-65.8] | 98.8 [98.5-98.9] | NaN | NaN |
| Cardiomyopathy and myocarditis | 10 | 20.0 [16.8-39.9] | 99.5 [99.3-99.6] | 42.4 [10.9-163.9] | 0.80 [0.59-1.10] |
| Atrial fibrillation and flutter | 10 | 70.0 [51.8-77.1] | 99.6 [99.4-99.7] | 175.2 [85.6-358.4] | 0.30 [0.12-0.78] |
| Aortic aneurysm | 2 | 50.0 [29.3-70.7] | 99.5 [99.3-99.6] | 106.2 [24.0-470.4] | 0.50 [0.13-2.01] |
| Peripheral vascular disease | 9 | 66.7 [48.3-75.1] | 99.5 [99.3-99.6] | 141.2 [69.3-288.0] | 0.33 [0.13-0.84] |
| Endocarditis | 1 | 0.0 [0.0-79.3] | 99.6 [99.4-99.7] | NaN | NaN |
| **Chronic respiratory diseases** |  |  |  |  |  |
| Chronic obstructive pulmonary disease | 34 | 70.6 [61.0-76.0] | 99.7 [99.6-99.8] | 275.2 [127.3-595.0] | 0.18 [0.07-0.43] |
| Pneumoconiosis | 0 | NaN | 99.9 [99.7-99.9] | NaN | 0.18 [0.07-0.43] |
| Asthma | 40 | 92.5 [84.4-93.2] | 99.9 [99.7-99.9] | 839.6 [270.0-2610.5] | 0.18 [0.07-0.43] |
| Interstitial lung disease and pulmonary sarcoidosis | 0 | NaN | 99.8 [99.6-99.8] | NaN | 0.18 [0.07-0.43] |
| **Cirrhosis of the liver** | 23 | 82.6 [70.2-85.6] | 98.9 [98.7-99.1] | 78.1 [51.9-117.3] | 0.18 [0.07-0.43] |
| **Digestive diseases (except cirrhosis)** |  |  |  |  |  |
| Peptic ulcer disease | 5 | 40.0 [27.7-61.0] | 99.8 [99.7-99.8] | 220.6 [55.2-881.8] | 0.60 [0.29-1.23] |
| Gastritis and duodenitis | 1 | 100.0 [20.7-100.0] | 99.8 [99.7-99.8] | 552.4 [230.1-1326.1] | NaN |
| Appendicitis | 2 | 100.0 [34.2-100.0] | 99.8 [99.6-99.8] | 460.2 [206.9-1023.4] | NaN |
| Paralytic ileus and intestinal obstruction without hernia | 0 | NaN | 99.8 [99.6-99.8] | NaN | NaN |
| Inguinal or femoral hernia | 1 | 100.0 [20.7-100.0] | 99.8 [99.6-99.8] | 460.3 [207.0-1023.8] | NaN |
| Non-infective inflammatory bowel disease | 9 | 100.0 [70.1-100.0] | 99.8 [99.6-99.8] | 459.0 [206.4-1020.8] | NaN |
| Vascular disorders of intestine | 1 | 0.0 [0.0-79.3] | 99.8 [99.7-99.8] | NaN | NaN |
| Gall bladder and bile duct disease | 4 | 50.0 [32.1-67.9] | 99.7 [99.6-99.8] | 197.1 [57.7-672.8] | 0.50 [0.19-1.34] |
| Pancreatitis | 2 | 100.0 [34.2-100.0] | 99.8 [99.6-99.8] | 460.2 [206.9-1023.4] | NaN |
| **Neurological disorders** |  |  |  |  |  |
| Alzheimer's disease and other dementias | 22 | 81.8 [69.1-85.1] | 99.3 [99.0-99.4] | 112.1 [69.5-181.0] | 0.18 [0.08-0.44] |
| Parkinson's disease | 17 | 94.1 [79.4-92.6] | 99.3 [99.1-99.4] | 136.0 [85.6-216.2] | 0.06 [0.01-0.40] |
| Epilepsy | 15 | 86.7 [70.5-87.9] | 99.4 [99.2-99.5] | 140.1 [83.8-234.2] | 0.13 [0.04-0.49] |
| Multiple sclerosis | 24 | 95.8 [84.5-94.5] | 99.3 [99.1-99.4] | 145.8 [91.3-232.8] | 0.04 [0.01-0.29] |
| Migraine | 7 | 85.7 [60.6-85.5] | 99.4 [99.2-99.5] | 139.0 [79.2-243.8] | 0.14 [0.02-0.88] |
| Tension-type headache | 0 | NaN | 99.3 [99.1-99.4] | NaN | NaN |
| **Mental and behavioral disorders** |  |  |  |  |  |
| Schizophrenia | 49 | 100.0 [92.7-100.0] | 99.7 [99.6-99.8] | 387.7 [185.0-812.5] | NaN |
| Alcohol use disorders | 13 | 76.9 [60.0-81.5] | 99.8 [99.7-99.8] | 423.1 [167.8-1066.9] | 0.23 [0.09-0.62] |
| Drug use disorders | 21 | 81.0 [67.9-84.4] | 99.9 [99.7-99.9] | 554.9 [203.9-1510.0] | 0.19 [0.08-0.46] |
| Major depressive disorder | 15 | 80.0 [64.2-83.6] | 99.5 [99.3-99.6] | 157.0 [87.9-280.6] | 0.20 [0.07-0.55] |
| Dysthymia | 0 | NaN | 99.2 [98.9-99.3] | NaN | NaN |
| Bipolar affective disorder | 13 | 76.9 [60.0-81.5] | 99.8 [99.6-99.8] | 352.6 [150.2-827.3] | 0.23 [0.09-0.62] |
| Anxiety disorders | 17 | 94.1 [79.4-92.6] | 99.6 [99.4-99.7] | 235.0 [128.7-428.8] | 0.06 [0.01-0.40] |
| Eating disorders | 2 | 50.0 [29.3-70.7] | 100.0 [99.9-100.0] | NaN | 0.50 [0.13-2.00] |
| Autism | 2 | 100.0 [34.2-100.0] | 99.9 [99.8-99.9] | 1380.5 [345.4-5517.1] | NaN |
| Asperger's syndrome | 0 | NaN | 100.0 [99.9-100.0] | NaN | NaN |
| Attention-deficit hyperactivity disorder | 2 | 100.0 [34.2-100.0] | 100.0 [99.9-100.0] | NaN | NaN |
| Conduct disorder | 0 | NaN | 99.9 [99.8-99.9] | NaN | NaN |
| Idiopathic intellectual disability | 1 | 0.0 [0.0-79.3] | 100.0 [99.8-99.9] | NaN | NaN |
| **Diabetes, urinary diseases and male infertility** |  |  |  |  |  |
| Diabetes mellitus | 202 | 87.1 [84.1-88.8] | 99.6 [99.4-99.6] | 202.9 [112.2-366.7] | 0.13 [0.09-0.19] |
| Acute glomerulonephritis | 0 | NaN | 99.9 [99.7-99.9] | NaN | NaN |
| Chronic kidney diseases | 29 | 48.3 [39.8-57.2] | 98.0 [97.7-98.2] | 24.0 [15.2-38.0] | 0.53 [0.37-0.75] |
| Tubulointerstitial nephritis, pyelonephritis, and urinary tract infections | 4 | 100.0 [51.0-100.0] | 99.8 [99.7-99.8] | 551.8 [229.9-1324.7] | NaN |
| Urolithiasis | 1 | 100.0 [20.7-100.0] | 99.8 [99.6-99.8] | 460.3 [207.0-1023.8] | NaN |
| Benign prostatic hyperplasia | 4 | 100.0 [51.0-100.0] | 99.8 [99.6-99.8] | 459.8 [206.8-1022.6] | NaN |
| Male infertility | 1 | 100.0 [20.7-100.0] | 99.8 [99.6-99.8] | 460.3 [207.0-1023.8] | NaN |
| **Gynecological diseases** |  |  |  |  |  |
| Uterine fibroids | 1 | 100.0 [20.7-100.0] | 99.9 [99.8-99.9] | 1381.0 [345.6-5519.1] | NaN |
| Polycystic ovarian syndrome | 0 | NaN | 100.0 [99.8-99.9] | NaN | NaN |
| Female infertility | 6 | 16.7 [16.1-43.3] | 99.8 [99.7-99.8] | 91.9 [12.5-673.7] | 0.83 [0.58-1.19] |
| Endometriosis | 2 | 100.0 [34.2-100.0] | 99.9 [99.7-99.9] | 690.3 [259.2-1837.8] | NaN |
| Genital prolapse | 0 | NaN | 99.8 [99.7-99.8] | NaN | NaN |
| Premenstrual syndrome | 0 | NaN | 100.0 [99.9-100.0] | NaN | NaN |
| **Hemoglobinopathies and hemolytic anemias** |  |  |  |  |  |
| Thalassemias | 14 | 42.9 [32.7-56.1] | 99.9 [99.8-99.9] | 589.1 [129.9-2671.1] | 0.57 [0.36-0.90] |
| Sickle cell disorders | 14 | 14.3 [12.8-31.1] | 100.0 [99.8-99.9] | 392.7 [37.7-4086.3] | 0.86 [0.69-1.06] |
| G6PD deficiency | 14 | 0.0 [0.0-21.5] | 100.0 [99.8-99.9] | NaN | NaN |
| **Musculoskeletal disorders** |  |  |  |  |  |
| Rheumatoid arthritis | 45 | 88.9 [81.1-90.6] | 99.6 [99.4-99.7] | 241.6 [129.0-452.4] | 0.11 [0.05-0.25] |
| Osteoarthritis | 40 | 70.0 [61.3-75.2] | 100.0 [99.8-99.9] | 1906.1 [265.8-13669.1] | 0.30 [0.19-0.48] |
| Low back pain | 13 | 92.3 [74.5-90.8] | 99.7 [99.5-99.7] | 317.3 [156.1-645.1] | 0.08 [0.01-0.51] |
| Neck pain | 3 | 33.3 [24.0-61.3] | 100.0 [99.8-99.9] | 920.0 [73.3-11549.5] | 0.67 [0.30-1.48] |
| Gout | 4 | 50.0 [32.1-67.9] | 99.6 [99.4-99.7] | 137.9 [43.3-439.6] | 0.50 [0.19-1.34] |
| **Congenital anomalies** | 23 | 95.7 [84.0-94.3] | 98.3 [98.0-98.5] | 55.8 [41.5-75.0] | 0.04 [0.01-0.30] |
| **Skin and subcutaneous diseases** |  |  |  |  |  |
| Eczema | 3 | 33.3 [24.0-61.3] | 99.9 [99.8-99.9] | 460.0 [55.4-3819.6] | 0.67 [0.30-1.48] |
| Psoriasis | 5 | 80.0 [52.0-82.0] | 100.0 [99.9-100.0] | NaN | 0.20 [0.03-1.15] |
| Cellulitis | 3 | 0.0 [0.0-56.1] | 100.0 [99.9-100.0] | NaN | NaN |
| Abscess, impetigo, and other bacterial skin diseases | 2 | 100.0 [34.2-100.0] | 99.7 [99.6-99.8] | 394.4 [188.2-826.6] | NaN |
| Scabies | 0 | NaN | 100.0 [99.9-100.0] | NaN | NaN |
| Fungal skin diseases | 1 | 100.0 [20.7-100.0] | 99.8 [99.6-99.8] | 460.3 [207.0-1023.8] | NaN |
| Viral skin diseases | 0 | NaN | 100.0 [99.8-99.9] | NaN | NaN |
| Acne vulgaris | 0 | NaN | 100.0 [99.8-99.9] | NaN | NaN |
| Alopecia areata | 1 | 100.0 [20.7-100.0] | 100.0 [99.8-99.9] | 2762.0 [389.2-19600.7] | NaN |
| Pruritus | 0 | NaN | 100.0 [99.9-100.0] | NaN | NaN |
| Urticaria | 1 | 100.0 [20.7-100.0] | 100.0 [99.9-100.0] | NaN | NaN |
| Decubitus ulcer | 0 | NaN | 100.0 [99.8-99.9] | NaN | NaN |
| **Sense organ diseases** |  |  |  |  |  |
| Glaucoma | 16 | 100.0 [80.6-100.0] | 99.9 [99.8-99.9] | 1373.5 [343.7-5489.1] | NaN |
| Cataracts | 12 | 91.7 [72.9-90.2] | 100.0 [99.8-99.9] | 2521.8 [352.7-18028.8] | 0.08 [0.01-0.54] |
| Macular degeneration | 18 | 100.0 [82.4-100.0] | 100.0 [99.8-99.9] | 2745.0 [386.8-19480.0] | NaN |
| Refraction and accommodation disorders | 4 | 50.0 [32.1-67.9] | 100.0 [99.8-99.9] | 1379.5 [154.2-12338.3] | 0.50 [0.19-1.33] |
| **Oral disorders** |  |  |  |  |  |
| Dental caries | 1 | 0.0 [0.0-79.3] | 99.8 [99.6-99.8] | NaN | NaN |
| Periodontal disease | 4 | 50.0 [32.1-67.9] | 99.8 [99.7-99.8] | 275.9 [74.1-1026.9] | 0.50 [0.19-1.33] |
| Edentulism | 3 | 0.0 [0.0-56.1] | 100.0 [99.9-100.0] | NaN | NaN |
| **Sudden infant death syndrome** | 0 | NaN | 100.0 [99.9-100.0] | NaN | NaN |
| **Injuries** |  |  |  |  |  |
| Road injury | 3 | 0.0 [0.0-56.1] | 99.9 [99.8-99.9] | NaN | NaN |
| Falls | 7 | 14.3 [14.5-39.4] | 100.0 [99.9-100.0] | NaN | 0.86 [0.63-1.16] |
| Drowning | 0 | NaN | 100.0 [99.9-100.0] | NaN | NaN |
| Fire, heat and hot substances | 3 | 66.7 [38.7-76.0] | 100.0 [99.9-100.0] | NaN | 0.33 [0.07-1.65] |
| Poisonings | 0 | NaN | 99.9 [99.8-99.9] | NaN | NaN |
| Exposure to mechanical forces | 2 | 0.0 [0.0-65.8] | 99.9 [99.8-99.9] | NaN | NaN |
| Adverse effects of medical treatment | 35 | 8.6 [7.7-17.6] | 99.6 [99.4-99.6] | 19.5 [5.8-66.0] | 0.92 [0.83-1.02] |
| Animal contact | 0 | NaN | 100.0 [99.9-100.0] | NaN | NaN |
| Self-harm | 4 | 25.0 [20.6-53.9] | 100.0 [99.9-100.0] | NaN | 0.75 [0.43-1.32] |
| Interpersonal violence | 0 | NaN | 99.9 [99.8-99.9] | NaN | NaN |
| Exposure to forces of nature | 1 | 0.0 [0.0-79.3] | 100.0 [99.9-100.0] | NaN | NaN |
| Collective violence and legal intervention | 0 | NaN | 100.0 [99.9-100.0] | NaN | NaN |
| **No GBD category** | 484 | 71.3 [69.1-73.2] | 91.4 [90.7-91.9] | 8.3 [7.2-9.6] | 0.31 [0.27-0.36] |

Number of trials per GBD category from the test set of 2,763 clinical trials. Sensitivities, specificities (in %) and likelihood ratios for each of the 171 GBD categories plus the “No GBD” category for the classifier using the Word Sense Disambiguation, the expert-based enrichment database and the priority to the health condition field.

**Table S3: Performances of the eight versions of the classifier to the 171 GBD categories**

| Word Sense Disambiguation | Expert-based enrichment | Priority to health condition field | Proportion of trials with correct automatic classification | | | | | Weighted average across GBD categories | |
| --- | --- | --- | --- | --- | --- | --- | --- | --- | --- |
|  |  |  | All trials N=2763 | One GBD category N=2092 | Two or more GBD categories N=187 | No GBD category N=484 | Sensitivity | | Specificity |
| Yes | Yes | Yes | 74.0 | 77.4 | 43.3 | 71.3 | 75.2 | | 98.0 |
| Yes | Yes | No | 74.0 | 77.4 | 44.4 | 70.5 | 75.2 | | 98.0 |
| Yes | No | Yes | 67.1 | 68.4 | 36.9 | 73.6 | 67.3 | | 97.0 |
| Yes | No | No | 67.8 | 69.3 | 38.5 | 72.7 | 68.6 | | 97.1 |
| No | Yes | Yes | 72.3 | 75.2 | 42.2 | 71.3 | 74.5 | | 97.8 |
| No | Yes | No | 72.2 | 75.2 | 43.3 | 70.5 | 74.5 | | 97.8 |
| No | No | Yes | 65.7 | 66.6 | 35.3 | 73.6 | 66.6 | | 96.8 |
| No | No | No | 66.3 | 67.5 | 36.9 | 72.7 | 67.9 | | 96.9 |

Exact-matching and weighted averaged sensitivities and specificities for eight versions of the classifier to the 171 GBD categories. Exact-matching corresponds to the proportion (in %) of trials for which the automatic GBD classification is correct. Exact-matching was estimated over all trials (N=2,763), over trials concerning a unique GBD category (N=2,092), over trials concerning two or more GBD categories (N=187), and over trials not relevant for the GBD (N=484). The weighted averaged sensitivity and specificity corresponds to the weighted average across GBD categories of the sensitivities and specificities of each GBD category plus the “No GBD” category (in %). The eight versions correspond to the combinations of: the use or not of the Word Sense Disambiguation during the text annotation, the use or not of the expert-based enrichment database, and the use or not of the priority to the health condition field as a prioritization rule.

**Table S5:** **Per-category evaluation of the performance of the baseline for the 28 GBD categories plus the “No GBD” category**

|  |  | Condition field | | Public title | | Scientific title | | All text fields | |
| --- | --- | --- | --- | --- | --- | --- | --- | --- | --- |
| GBD category | Nb | Sensitivity | Specificity | Sensitivity | Specificity | Sensitivity | Specificity | Sensitivity | Specificity |
| Neoplasms | 958 | 46.3  [44.8-48.0] | 99.6  [99.4-99.7] | 33.1  [31.6-34.7] | 100.0  [99.8-100.0] | 31.6  [30.2-33.2] | 100.0  [99.8-100.0] | 49.8  [48.2-51.4] | 99.6  [99.4-99.7] |
| Diabetes, urinary diseases and male infertility | 242 | 37.2  [34.3-40.5] | 99.8  [99.6-99.8] | 14.5  [12.8-17.3] | 99.9  [99.8-99.9] | 18.6  [16.6-21.6] | 99.8  [99.7-99.9] | 41.7  [38.7-45.0] | 99.7  [99.5-99.8] |
| Cardiovascular and circulatory diseases | 235 | 4.3  [3.6-6.4] | 100.0  [99.8-100.0] | 2.1  [1.9-3.9] | 100.0  [99.8-100.0] | 2.6  [2.2-4.4] | 100.0  [99.8-100.0] | 5.5  [4.7-7.8] | 100.0  [99.8-100.0] |
| Mental and behavioral disorders | 143 | 32.9  [29.4-37.2] | 100.0  [99.8-100.0] | 26.6  [23.5-30.8] | 100.0  [99.8-100.0] | 26.6  [23.5-30.8] | 100.0  [99.8-100.0] | 40.6  [36.8-44.9] | 100.0  [99.8-100.0] |
| Musculoskeletal disorders | 113 | 63.7  [58.8-67.7] | 100.0  [99.8-100.0] | 39.8  [35.6-44.7] | 100.0  [99.8-100.0] | 42.5  [38.2-47.3] | 100.0  [99.8-100.0] | 66.4  [61.5-70.2] | 99.9  [99.8-99.9] |
| HIV/AIDS | 97 | 86.6  [81.7-88.7] | 99.8  [99.6-99.8] | 53.6  [48.5-58.4] | 99.7  [99.6-99.8] | 63.9  [58.6-68.2] | 99.6  [99.4-99.7] | 91.8  [87.3-93.0] | 99.5  [99.3-99.6] |
| Neurological disorders | 93 | 52.7  [47.5-57.7] | 100.0  [99.9-100.0] | 37.6  [33.2-43.1] | 100.0  [99.9-100.0] | 35.5  [31.2-40.9] | 100.0  [99.9-100.0] | 55.9  [50.6-60.7] | 100.0  [99.9-100.0] |
| Chronic respiratory diseases | 81 | 67.9  [62.0-72.2] | 100.0  [99.8-100.0] | 54.3  [48.7-59.5] | 100.0  [99.9-100.0] | 46.9  [41.6-52.5] | 100.0  [99.9-100.0] | 76.5  [70.7-80.0] | 100.0  [99.8-100.0] |
| Sense organ diseases | 56 | 55.4  [48.6-61.4] | 100.0  [99.9-100.0] | 32.1  [27.2-39.4] | 100.0  [99.9-100.0] | 41.1  [35.3-48.0] | 99.9  [99.8-99.9] | 58.9  [52.0-64.7] | 99.9  [99.8-99.9] |
| Injuries | 56 | 8.9  [7.6-15.5] | 99.9  [99.8-99.9] | 7.1  [6.3-13.5] | 100.0  [99.9-100.0] | 3.6  [3.7-9.4] | 100.0  [99.8-100.0] | 12.5  [10.5-19.4] | 99.9  [99.7-99.9] |
| Diarrhea, lower respiratory infections, meningitis, and other common infectious diseases | 49 | 40.8  [34.7-48.3] | 99.9  [99.7-99.9] | 24.5  [20.4-32.3] | 99.9  [99.7-99.9] | 24.5  [20.4-32.3] | 99.9  [99.7-99.9] | 44.9  [38.4-52.1] | 99.8  [99.7-99.8] |
| Maternal disorders | 43 | 9.3  [8.1-17.2] | 100.0  [99.9-100.0] | 9.3  [8.1-17.2] | 100.0  [99.9-100.0] | 7.0  [6.4-14.6] | 100.0  [99.9-100.0] | 9.3  [8.1-17.2] | 100.0  [99.9-100.0] |
| Digestive diseases (except cirrhosis) | 32 | 12.5  [10.6-22.4] | 100.0  [99.8-100.0] | 3.1  [4.3-12.0] | 100.0  [99.9-100.0] | 3.1  [4.3-12.0] | 100.0  [99.9-100.0] | 12.5  [10.6-22.4] | 100.0  [99.8-99.9] |
| Cirrhosis of the liver | 23 | 0.0  [0.0-14.3] | 100.0  [99.9-100.0] | 0.0  [0.0-14.3] | 100.0  [99.9-100.0] | 0.0  [0.0-14.3] | 100.0  [99.9-100.0] | 0.0  [0.0-14.3] | 100.0  [99.9-100.0] |
| Congenital anomalies | 23 | 0.0  [0.0-14.3] | 100.0  [99.9-100.0] | 0.0  [0.0-14.3] | 100.0  [99.9-100.0] | 0.0  [0.0-14.3] | 100.0  [99.9-100.0] | 0.0  [0.0-14.3] | 100.0  [99.9-100.0] |
| Skin and subcutaneous diseases | 22 | 36.4  [28.9-47.9] | 100.0  [99.9-100.0] | 31.8  [25.3-43.8] | 100.0  [99.9-100.0] | 31.8  [25.3-43.8] | 100.0  [99.9-100.0] | 36.4  [28.9-47.9] | 100.0  [99.9-100.0] |
| Hepatitis | 17 | 82.4  [67.5-85.3] | 99.7  [99.5-99.7] | 70.6  [56.6-77.0] | 99.9  [99.7-99.9] | 58.8  [46.4-68.0] | 99.9  [99.7-99.9] | 88.2  [73.3-89.1] | 99.6  [99.4-99.7] |
| Tuberculosis | 16 | 93.8  [78.3-92.2] | 100.0  [99.8-99.9] | 75.0  [60.1-80.2] | 99.9  [99.8-99.9] | 62.5  [49.1-71.0] | 99.9  [99.8-99.9] | 93.8  [78.3-92.2] | 99.9  [99.7-99.9] |
| Nutritional deficiencies | 16 | 0.0  [0.0-19.4] | 100.0  [99.9-100.0] | 0.0  [0.0-19.4] | 100.0  [99.9-100.0] | 0.0  [0.0-19.4] | 100.0  [99.9-100.0] | 0.0  [0.0-19.4] | 100.0  [99.9-100.0] |
| Hemoglobinopathies and hemolytic anemias | 16 | 0.0  [0.0-19.4] | 100.0  [99.9-100.0] | 0.0  [0.0-19.4] | 100.0  [99.9-100.0] | 0.0  [0.0-19.4] | 100.0  [99.9-100.0] | 0.0  [0.0-19.4] | 100.0  [99.9-100.0] |
| Malaria | 14 | 100.0  [78.5-100.0] | 100.0  [99.9-100.0] | 71.4  [55.9-77.8] | 100.0  [99.9-100.0] | 85.7  [68.9-87.2] | 100.0  [99.8-100.0] | 100.0  [78.5-100.0] | 100.0  [99.8-100.0] |
| Gynecological diseases | 11 | 9.1  [10.5-28.9] | 99.9  [99.8-99.9] | 9.1  [10.5-28.9] | 100.0  [99.8-100.0] | 18.2  [15.6-37.3] | 100.0  [99.8-100.0] | 18.2  [15.6-37.3] | 99.9  [99.8-99.9] |
| Neonatal disorders | 10 | 0.0  [0.0-27.8] | 100.0  [99.9-100.0] | 0.0  [0.0-27.8] | 100.0  [99.9-100.0] | 0.0  [0.0-27.8] | 100.0  [99.9-100.0] | 0.0  [0.0-27.8] | 100.0  [99.9-100.0] |
| Oral disorders | 8 | 37.5  [27.3-55.8] | 100.0  [99.8-100.0] | 25.0  [19.9-46.4] | 100.0  [99.9-100.0] | 12.5  [13.2-36.1] | 100.0  [99.9-100.0] | 50.0  [35.5-64.5] | 100.0  [99.8-100.0] |
| Neglected tropical diseases excluding malaria | 7 | 42.9  [30.3-60.5] | 100.0  [99.8-100.0] | 57.1  [39.5-69.7] | 100.0  [99.8-100.0] | 57.1  [39.5-69.7] | 100.0  [99.9-100.0] | 57.1  [39.5-69.7] | 100.0  [99.8-100.0] |
| Leprosy | 2 | 100.0  [34.2-100.0] | 100.0  [99.8-100.0] | 100.0  [34.2-100.0] | 100.0  [99.9-100.0] | 100.0  [34.2-100.0] | 100.0  [99.9-100.0] | 100.0  [34.2-100.0] | 100.0  [99.8-100.0] |
| Sexually transmitted diseases excluding HIV | 1 | 0.0  [0.0-79.3] | 100.0  [99.9-100.0] | 0.0  [0.0-79.3] | 100.0  [99.9-100.0] | 0.0  [0.0-79.3] | 100.0  [99.9-100.0] | 0.0  [0.0-79.3] | 100.0  [99.9-100.0] |
| Sudden infant death syndrome | 0 | NaN  [0.0-100.0] | 100.0 [99.9-100.0] | NaN  [0.0-100.0] | 100.0  [99.9-100.0] | NaN  [0.0-100.0] | 100.0  [99.9-100.0] | NaN  [0.0-100.0] | 100.0  [99.9-100.0] |
| No GBD category | 407 | 98.5  [97.4-98.7] | 42.3  [41.3-43.3] | 100.0  [99.1-100.0] | 28.7  [27.8-29.7] | 99.3  [98.3-99.3] | 28.7  [27.8-29.7] | 97.8  [96.6-98.1] | 46.1  [45.1-47.2] |

Number of trials per GBD category from the test set of 2,763 clinical trials. Sensitivities and specificities (in %) of the 28 GBD categories plus the “No GBD” category for the classification of clinical trial records towards GBD categories without using the UMLS knowledge source but based on the recognition in free text of the names of diseases defining in each GBD category only. For the baseline a clinical trial records was classified with a GBD category if at least one of the 291 disease names from the GBD cause list defining that GBD category appeared verbatim in the condition field, the public or scientific titles, separately, or in at least one of these three text fields.

**Dataset S1: Expert-based enrichment database for the classification to the 28 GBD categories.**

| CUI | Concept_name | GBD_classification | CUI | Concept_name | GBD_classification |
| --- | --- | --- | --- | --- | --- |
| C0000737 | Abdominal Pain | No GBD | C0242184 | Hypoxia | No GBD |
| C0000809 | Abortion, Habitual | Maternal disorders | C0242339 | Dyslipidemias | No GBD |
| C0000924 | Accidents | No GBD | C0242429 | Sore Throat | No GBD |
| C0000925 | Incised wound | No GBD | C0242488 | Acute Lung Injury | No GBD |
| C0001122 | Acidosis | No GBD | C0242510 | Drug usage | Mental and behavioral disorders |
| C0001125 | Acidosis, Lactic | No GBD | C0242606 | Oxidative Stress | No GBD |
| C0001314 | Acute Disease | No GBD | C0242781 | disease transmission | No GBD |
| C0002351 | Altitude Sickness | No GBD | C0242966 | Systemic Inflammatory Response Syndrome | No GBD |
| C0002690 | Amputation Stumps | No GBD | C0242992 | Multiple Chemical Sensitivity | No GBD |
| C0002726 | Amyloidosis | No GBD | C0243001 | Abdominal Abscess | No GBD |
| C0002792 | anaphylaxis | No GBD | C0243026 | Sepsis | No GBD |
| C0002994 | Angioedema | No GBD | C0243083 | associated disease | No GBD |
| C0003123 | Anorexia | No GBD | C0243088 | sequelae aspects | No GBD |
| C0003578 | Apnea | No GBD | C0262593 | Peripheral Nerve Injuries | No GBD |
| C0003782 | Argyria | No GBD | C0262627 | Seroma | No GBD |
| C0003794 | Arm Injuries | No GBD | C0264490 | Acute respiratory failure | No GBD |
| C0004144 | Atelectasis | No GBD | C0264492 | Chronic respiratory failure | No GBD |
| C0004368 | Autoimmunity | No GBD | C0264886 | Conduction disorder of the heart | No GBD |
| C0004610 | Bacteremia | No GBD | C0264995 | Occlusion of artery (disorder) | No GBD |
| C0004623 | Bacterial Infections | No GBD | C0266836 | Infantile Colic | No GBD |
| C0004930 | Behavior Disorders | No GBD | C0267716 | Incisional hernia | No GBD |
| C0004936 | Mental disorders | No GBD | C0268064 | Transfusion hemosiderosis | No GBD |
| C0005658 | bite injury | No GBD | C0268381 | Primary amyloidosis | No GBD |
| C0006107 | Brain Concussion | No GBD | C0268407 | Senile cardiac amyloidosis | No GBD |
| C0006114 | Cerebral Edema | No GBD | C0268575 | Isovaleryl-CoA dehydrogenase deficiency | No GBD |
| C0006434 | Burn injury | Injuries | C0270327 | Bedwetting | No GBD |
| C0006625 | Cachexia | No GBD | C0270805 | Hemiplegic cerebral palsy | No GBD |
| C0007222 | Cardiovascular Diseases | No GBD | C0271650 | Impaired glucose tolerance | Diabetes, urinary diseases and male infertility |
| C0007789 | Cerebral Palsy | No GBD | C0274281 | Injury due to exposure to external cause | No GBD |
| C0008031 | Chest Pain | No GBD | C0275551 | Primary bacterial peritonitis | No GBD |
| C0008039 | Cheyne-Stokes Respiration | No GBD | C0277556 | Recurrent disease | No GBD |
| C0008679 | Chronic disease | No GBD | C0277562 | Adult disease | No GBD |
| C0008715 | Chronically Ill | No GBD | C0277564 | Acquired disease | No GBD |
| C0008767 | Cicatrization | No GBD | C0278139 | Moderate pain | No GBD |
| C0009421 | Comatose | No GBD | C0281856 | Generalized aches and pains | No GBD |
| C0009450 | Communicable Diseases | No GBD | C0282193 | Iron Overload | No GBD |
| C0009492 | Compartment syndromes | No GBD | C0282666 | Very Low Birth Weight | No GBD |
| C0009566 | Complication | No GBD | C0332448 | Infiltration | No GBD |
| C0009814 | Acquired stenosis | No GBD | C0332568 | Pad Mass | No GBD |
| C0009938 | Contusions | No GBD | C0332679 | Crushing injury | Injuries |
| C0010200 | Coughing | No GBD | C0332687 | Blisters, epidermal loss [second degree], unspecified site | No GBD |
| C0010340 | Critical Illness | No GBD | C0332798 | Open wound | No GBD |
| C0010674 | Cystic Fibrosis | No GBD | C0332803 | Surgical wound | No GBD |
| C0010957 | Tissue damage | No GBD | C0332837 | Traumatic implants | No GBD |
| C0011164 | Abnormal degeneration | No GBD | C0332840 | Amputated structure (morphologic abnormality) | No GBD |
| C0011168 | Deglutition Disorders | No GBD | C0332853 | Anastomosis | No GBD |
| C0011175 | Dehydration | No GBD | C0333262 | Vesicle (morphologic abnormality) | No GBD |
| C0011560 | Amyloid deposition | No GBD | C0333641 | Atrophic | No GBD |
| C0011880 | Diabetic Ketoacidosis | Diabetes, urinary diseases and male infertility | C0334094 | Proliferation (morphologic abnormality) | No GBD |
| C0011881 | Diabetic Nephropathy | Diabetes, urinary diseases and male infertility | C0338596 | Spastic cerebral palsy | No GBD |
| C0011884 | Diabetic Retinopathy | No GBD | C0338656 | Impaired cognition | No GBD |
| C0012634 | Disease | No GBD | C0340861 | Electromechanical dissociation | No GBD |
| C0012739 | Disseminated Intravascular Coagulation | No GBD | C0341539 | Parastomal hernia NOS | No GBD |
| C0012833 | Dizziness | No GBD | C0342257 | Complications of Diabetes Mellitus | Diabetes, urinary diseases and male infertility |
| C0013146 | Drug abuse | Mental and behavioral disorders | C0342751 | Generalized glycogen storage disease of infants | No GBD |
| C0013404 | Dyspnea | No GBD | C0342895 | Fish-Eye Disease | No GBD |
| C0013604 | Edema | No GBD | C0343641 | Human papilloma virus infection | No GBD |
| C0013687 | effusion | No GBD | C0344307 | Absence of pain sensation | No GBD |
| C0013922 | Embolism | No GBD | C0346153 | Breast Cancer, Familial | Neoplasms |
| C0014009 | Empyema | No GBD | C0376297 | Cardiac Death | No GBD |
| C0014591 | Epistaxis | No GBD | C0392164 | Pulmonary Cystic Fibrosis | No GBD |
| C0015230 | Exanthema | No GBD | C0392615 | Adverse effect of radiation therapy | Injuries |
| C0015376 | Extravasation | No GBD | C0392618 | Postoperative infection | No GBD |
| C0015672 | Fatigue | No GBD | C0392707 | Atopy | No GBD |
| C0015674 | Chronic Fatigue Syndrome | No GBD | C0398353 | Hypercapnic respiratory failure | No GBD |
| C0015732 | Fecal Incontinence | No GBD | C0398650 | Immune thrombocytopenic purpura | No GBD |
| C0015802 | Fracture of femur, part unspecified | No GBD | C0403447 | Chronic Kidney Insufficiency | Diabetes, urinary diseases and male infertility |
| C0015806 | Fracture of neck of femur | No GBD | C0409312 | Rupture of anterior cruciate ligament | No GBD |
| C0016059 | Fibrosis | No GBD | C0410158 | Muscle damage | No GBD |
| C0016204 | Flatulence | No GBD | C0410256 | muscle injury | No GBD |
| C0016382 | Flushing | No GBD | C0413252 | Hypothermia due to exposure | No GBD |
| C0016385 | Cardiac Flutter | Cardiovascular and circulatory diseases | C0426070 | Flexed fetal attitude | No GBD |
| C0016470 | Food Allergy | No GBD | C0427149 | Gait, Drop Foot | No GBD |
| C0016658 | Fracture | No GBD | C0428908 | Sinus Node Dysfunction (disorder) | No GBD |
| C0016662 | Fracture of unspecified bone, open | No GBD | C0428977 | Bradycardia | No GBD |
| C0017181 | Gastrointestinal hemorrhage, unspecified | No GBD | C0439857 | Dependence | No GBD |
| C0018133 | Graft-vs-Host Disease | Injuries | C0443146 | Autoimmune reaction | No GBD |
| C0018273 | Growth Disorders | No GBD | C0443306 | Spastic | No GBD |
| C0018482 | Haemophilus Infections | No GBD | C0449430 | Physiological Stress | No GBD |
| C0018520 | Halitosis | No GBD | C0452131 | Effects of vibration | No GBD |
| C0018674 | Unspecified injury of head | No GBD | C0474368 | Labor Pain | No GBD |
| C0018681 | Headache | No GBD | C0476273 | Respiratory distress | No GBD |
| C0018790 | Cardiac Arrest | No GBD | C0481391 | Lifestyle-related condition | No GBD |
| C0018799 | Heart Diseases | No GBD | C0496118 | Effects of electric current | No GBD |
| C0018801 | Heart failure | No GBD | C0497406 | Overweight | No GBD |
| C0018802 | Congestive heart failure | No GBD | C0520817 | Physical disability | No GBD |
| C0018834 | Heartburn | No GBD | C0520893 | Localized desquamation | No GBD |
| C0018944 | Hematoma | No GBD | C0520904 | Postoperative Nausea | No GBD |
| C0018991 | Hemiplegia, unspecified | No GBD | C0520905 | Vomiting, Postoperative | No GBD |
| C0019054 | Hemolysis (disorder) | No GBD | C0520909 | Postoperative Nausea and Vomiting | No GBD |
| C0019247 | Hereditary Diseases | No GBD | C0524620 | Metabolic Syndrome X | No GBD |
| C0019322 | Umbilical hernia | No GBD | C0544885 | Mutation, Nonsense | No GBD |
| C0019326 | Ventral Hernia | No GBD | C0546255 | Colostomy site | No GBD |
| C0019557 | Hip Fractures | No GBD | C0546256 | Gastrostomy site | No GBD |
| C0020162 | Fracture of humerus | No GBD | C0546817 | Fluid overload | No GBD |
| C0020175 | Hunger | No GBD | C0559307 | Breathy voice quality | No GBD |
| C0020429 | Hyperalgesia | No GBD | C0559469 | Allergy to eggs | No GBD |
| C0020443 | Hypercholesterolemia | No GBD | C0559470 | Allergy to peanuts | No GBD |
| C0020456 | Hyperglycemia | Diabetes, urinary diseases and male infertility | C0574785 | Lower Urinary Tract Symptoms | No GBD |
| C0020459 | Hyperinsulinism | No GBD | C0575090 | Equilibration disorder | No GBD |
| C0020488 | Hypernatremia | No GBD | C0577573 | Mass of body region | No GBD |
| C0020505 | Hyperphagia | No GBD | C0599156 | Transition Mutation | No GBD |
| C0020507 | Hyperplasia | No GBD | C0599766 | Recovery of Function | No GBD |
| C0020517 | Hypersensitivity | No GBD | C0600142 | Hot flushes | No GBD |
| C0020523 | Immediate hypersensitivity | No GBD | C0600688 | Toxic effect | No GBD |
| C0020564 | Hypertrophy | No GBD | C0677660 | Emotional problems | No GBD |
| C0020615 | Hypoglycemia | No GBD | C0677932 | Progressive Neoplastic Disease | Neoplasms |
| C0020963 | Immune Tolerance | No GBD | C0678236 | Rare Diseases | No GBD |
| C0021053 | Immune System Diseases | No GBD | C0678356 | alcohol effect | No GBD |
| C0021167 | Incontinence | No GBD | C0679381 | Pediatric Disorder | No GBD |
| C0021368 | Inflammation | No GBD | C0679861 | Complications of treatment | Injuries |
| C0021655 | Insulin Resistance | Diabetes, urinary diseases and male infertility | C0683278 | Mental Suffering | No GBD |
| C0022346 | Icterus | No GBD | C0684309 | Disease model | No GBD |
| C0022660 | Kidney Failure, Acute | No GBD | C0684320 | Disease regression | No GBD |
| C0022661 | Kidney Failure, Chronic | Diabetes, urinary diseases and male infertility | C0684336 | Impaired health | No GBD |
| C0022744 | Knee Injuries | No GBD | C0694563 | Excessive daytime somnolence | No GBD |
| C0022951 | Lactose Intolerance | No GBD | C0699753 | Cancer Relapse | No GBD |
| C0023212 | Left-Sided Heart Failure | No GBD | C0700198 | Pulmonary aspiration | No GBD |
| C0023530 | Leukopenia | No GBD | C0700319 | Mentally ill chemical abuse | Mental and behavioral disorders |
| C0026771 | Unspecified multiple injuries | No GBD | C0700323 | Observation of Neuromuscular Block | No GBD |
| C0026821 | Muscle Cramp | No GBD | C0700361 | Emotional distress | No GBD |
| C0026837 | Muscle Rigidity | No GBD | C0728867 | Drug effect disorder | Mental and behavioral disorders |
| C0026838 | Muscle Spasticity | No GBD | C0730345 | Microalbuminuria | No GBD |
| C0027497 | Nausea | No GBD | C0740697 | Psychosocial problem | No GBD |
| C0027498 | Nausea and vomiting | No GBD | C0741282 | atrial fibrillation recurrent | Cardiovascular and circulatory diseases |
| C0027769 | Nervousness | No GBD | C0741923 | cardiac event | No GBD |
| C0027947 | Neutropenia | No GBD | C0742006 | Catheter infection | No GBD |
| C0028259 | Nodule | No GBD | C0751956 | Acute Cerebrovascular Accidents | Cardiovascular and circulatory diseases |
| C0028734 | Nocturia | No GBD | C0815107 | psychological distress | Mental and behavioral disorders |
| C0028754 | Obesity | No GBD | C0847097 | Heartburn acidity | No GBD |
| C0028778 | Obstruction | No GBD | C0848377 | trauma to the abdomen | No GBD |
| C0029443 | Osteomyelitis | No GBD | C0850624 | cardiovascular risk factor | No GBD |
| C0029944 | Drug Overdose | Mental and behavioral disorders | C0850758 | Pelvic pain female | No GBD |
| C0030193 | Pain | No GBD | C0852694 | Cartilage injury | No GBD |
| C0030200 | Pain, Intractable | No GBD | C0854135 | Pseudomonas aeruginosa infection | No GBD |
| C0030201 | Pain, Postoperative | No GBD | C0854706 | Infection of newborn NOS | Neonatal disorders |
| C0030486 | Paraplegia, unspecified | No GBD | C0857122 | hyponatremic | No GBD |
| C0030499 | Parasitic Diseases | No GBD | C0857496 | Thromboembolic event | No GBD |
| C0030794 | Pelvic Pain | No GBD | C0860239 | Catheter related infection | No GBD |
| C0031154 | Peritonitis, unspecified | No GBD | C0866323 | Severe toxemia | No GBD |
| C0032227 | Pleural effusion disorder | No GBD | C0867389 | Chronic graft-versus-host disease | Injuries |
| C0032320 | Pneumoperitoneum | No GBD | C0876926 | Traumatic Brain Injury | No GBD |
| C0032343 | Poisoning | Injuries | C0877008 | Enzyme inhibition disorder | No GBD |
| C0032787 | Postoperative Complications | No GBD | C0877248 | Adverse event | No GBD |
| C0032788 | Postoperative Hemorrhage | No GBD | C0877445 | Candidemia | No GBD |
| C0032962 | Pregnancy Complications | Maternal disorders | C0878787 | Growth failure | No GBD |
| C0033119 | Puncture wound | No GBD | C0879626 | Adverse effects | No GBD |
| C0034063 | Pulmonary Edema | No GBD | C0920269 | Microsatellite Instability | No GBD |
| C0034065 | Pulmonary Embolism | No GBD | C0920563 | Insulin Sensitivity | No GBD |
| C0034372 | Quadriplegia | No GBD | C0940933 | infection as a complication | No GBD |
| C0034535 | Radiation Syndrome | Injuries | C0948008 | Ischemic stroke | Cardiovascular and circulatory diseases |
| C0035078 | Kidney Failure | No GBD | C0949177 | White spot lesion | No GBD |
| C0035222 | Respiratory Distress Syndrome, Adult | No GBD | C1096106 | wound complication | No GBD |
| C0035229 | Respiratory Insufficiency | No GBD | C1110554 | Cardiovascular occlusion | Cardiovascular and circulatory diseases |
| C0035242 | Respiratory Tract Diseases | No GBD | C1112442 | Female sexual dysfunction | No GBD |
| C0036429 | Sclerosis | No GBD | C1135191 | Unspecified systolic (congestive) heart failure | No GBD |
| C0036572 | Seizures | No GBD | C1135196 | Unspecified diastolic (congestive) heart failure | No GBD |
| C0036690 | Septicemia | No GBD | C1135361 | Persistent pulmonary hypertension | No GBD |
| C0036877 | organic sexual dysfunction | No GBD | C1140999 | Contraction (finding) | No GBD |
| C0036974 | Shock | No GBD | C1142264 | critical limb ischemia | No GBD |
| C0036980 | Cardiogenic shock | No GBD | C1145670 | Respiratory Failure | No GBD |
| C0036982 | Shock, Hemorrhagic | No GBD | C1155266 | inflammatory response | No GBD |
| C0036983 | Septic Shock | No GBD | C1171258 | Complication Aspects | No GBD |
| C0037006 | Shoulder Fractures | No GBD | C1260922 | Unspecified abnormalities of breathing | No GBD |
| C0037052 | Sick Sinus Syndrome | No GBD | C1261287 | Stenosis | No GBD |
| C0037088 | Signs and Symptoms | No GBD | C1262018 | Graft failure | No GBD |
| C0037384 | Snoring | No GBD | C1265601 | Solitary mass | No GBD |
| C0037578 | Soft Tissue Injuries | No GBD | C1265679 | Wound, healed (morphologic abnormality) | No GBD |
| C0037929 | Spinal Cord Injuries | No GBD | C1265875 | Disintegration (morphologic abnormality) | No GBD |
| C0038443 | Stress, Psychological | No GBD | C1277187 | Left ventricular systolic dysfunction | No GBD |
| C0038454 | Cerebrovascular accident | Cardiovascular and circulatory diseases | C1291077 | Abdominal bloating | No GBD |
| C0038561 | Submersion | No GBD | C1298685 | Chronic pain syndrome | No GBD |
| C0038580 | Substance Dependence | Mental and behavioral disorders | C1306597 | Psychiatric problem | No GBD |
| C0038586 | Substance Use Disorders | Mental and behavioral disorders | C1313952 | Periodic breathing | No GBD |
| C0038587 | Substance Withdrawal Syndrome | Mental and behavioral disorders AND Injuries | C1368999 | Late effect of medical intervention | Injuries |
| C0038941 | Surgical Wound Infection | No GBD | C1373218 | Decreased Immunologic Activity [PE] | No GBD |
| C0039070 | Syncope | No GBD | C1392786 | Alteration Of Cognitive Function | No GBD |
| C0039082 | Syndrome | No GBD | C1397014 | Imbalance | No GBD |
| C0039231 | Tachycardia | No GBD | C1442161 | Gene Deletion Abnormality | No GBD |
| C0039504 | Tendon Injuries | No GBD | C1457887 | Symptoms | No GBD |
| C0040185 | Tibial Fractures | No GBD | C1504525 | Acute lymphoblastic leukemia recurrent | Neoplasms |
| C0040822 | Tremor | No GBD | C1510420 | Cavitation | No GBD |
| C0041755 | Adverse reaction to drug | Injuries | C1510432 | Radiation Sickness | Injuries |
| C0041909 | Upper gastrointestinal hemorrhage | No GBD | C1512441 | High-Risk Cancer | No GBD |
| C0042024 | Urinary Incontinence | No GBD | C1516986 | Evaluable Disease | No GBD |
| C0042510 | Ventricular Fibrillation | No GBD | C1517205 | Flare | No GBD |
| C0042749 | Viremia, unspecified | No GBD | C1521724 | Alzheimer's Disease Pathway KEGG | Neurological disorders |
| C0042963 | Vomiting | No GBD | C1527304 | Allergic Reaction | No GBD |
| C0043144 | Wheezing | No GBD | C1527311 | Brain Edema | No GBD |
| C0043145 | Whiplash Injuries | Injuries | C1533163 | Disorder of cellular component of blood | No GBD |
| C0043194 | Wiskott-Aldrich Syndrome | No GBD | C1559154 | Rash and Dermatitis Adverse Event Associated with Chemoradiation | Injuries |
| C0043241 | Wound Infection | No GBD | C1561642 | Chronic kidney disease, Stage V | Diabetes, urinary diseases and male infertility |
| C0043246 | Laceration | No GBD | C1561643 | Chronic Kidney Diseases | Diabetes, urinary diseases and male infertility |
| C0043250 | Injury wounds | No GBD | C1565249 | Mobility Limitation | No GBD |
| C0043251 | Wounds and Injuries | No GBD | C1566590 | Delayed Graft Function | No GBD |
| C0079487 | Helicobacter Infections | Digestive diseases (except cirrhosis) | C1609535 | Invasive Candidiasis | No GBD |
| C0085111 | Ankle Injuries | No GBD | C1705285 | Mutation Abnormality | No GBD |
| C0085281 | Addictive Behavior | Mental and behavioral disorders | C1705427 | Germline Mutation Abnormality | No GBD |
| C0085298 | Sudden Cardiac Death | No GBD | C1705759 | Gene Amplification Abnormality | No GBD |
| C0085426 | Gram-Positive Bacterial Infections | No GBD | C1709246 | Non-Neoplastic Disorder | No GBD |
| C0085580 | Essential Hypertension | No GBD | C1719672 | Severe Sepsis | No GBD |
| C0085584 | Encephalopathies | No GBD | C1838069 | SCHIZOPHRENIA 3 (disorder) | Mental and behavioral disorders |
| C0085612 | Ventricular arrhythmia | No GBD | C1848140 | COGNITIVE FUNCTION 1, SOCIAL | No GBD |
| C0085631 | Agitation | No GBD | C1850383 | Neuropathy, Painful | No GBD |
| C0085632 | Indifferent mood | No GBD | C1860404 | VITREORETINOPATHY, NEOVASCULAR INFLAMMATORY | No GBD |
| C0085633 | Mood swings | Mental and behavioral disorders | C1868649 | PANIC DISORDER 1 | Mental and behavioral disorders |
| C0087086 | Thrombus | No GBD | C1959609 | Erosion lesion | No GBD |
| C0087169 | Withdrawal Symptoms | Mental and behavioral disorders AND Injuries | C1962948 | Atelectasis Adverse Event | No GBD |
| C0149721 | Left Ventricular Hypertrophy | No GBD | C1963065 | Apnea Adverse Event | No GBD |
| C0149746 | Orthostasis | No GBD | C1963179 | Nausea Adverse Event | No GBD |
| C0150055 | Chronic pain | No GBD | C1998242 | Traumatic injury of skeletal muscle | No GBD |
| C0151293 | Chronic Headache | Neurological disorders | C2004489 | Regurgitation | No GBD |
| C0151517 | Complete atrioventricular block | No GBD | C2316810 | Chronic kidney disease stage 5 | Diabetes, urinary diseases and male infertility |
| C0151740 | Intracranial Hypertension | No GBD | C2349423 | Medication overuse headache | No GBD |
| C0155773 | Portal vein thrombosis | No GBD | C2609414 | Acute kidney injury | No GBD |
| C0156409 | Postmenopausal atrophic vaginitis | No GBD | C2712889 | Novel H1N1 influenza | No GBD |
| C0159658 | Fracture of clavicle | No GBD | C2729169 | Wrinkled structure | No GBD |
| C0159877 | Fracture of ankle | No GBD | C2745965 | Emergencies [Disease/Finding] | No GBD |
| C0160087 | Sprain of ankle, unspecified site | No GBD | C2825055 | Recurrence (disease attribute) | No GBD |
| C0160420 | Injury of kidney | No GBD | C2874046 | Type 1 diabetes mellitus with diabetic neuralgia | Diabetes, urinary diseases and male infertility |
| C0161479 | Injury of nerve(s) of unspecified body region | No GBD | C2882221 | Acute pulmonary embolism NOS | No GBD |
| C0161816 | Cardiac complications | No GBD | C2891275 | Cardiac allograft vasculopathy | No GBD |
| C0162566 | Porphyria Cutanea Tarda | No GBD | C2919062 | Shot (injury) | Injuries |
| C0162633 | Viral Shedding | No GBD | C2936258 | Peri-Implantitis | Oral disorders |
| C0184567 | Acute onset pain | No GBD | C2936380 | Neointima | No GBD |
| C0220981 | Metabolic acidosis | No GBD | C2936490 | Out-of-Hospital Cardiac Arrest | No GBD |
| C0221208 | Traumatic injection site | No GBD | C2938905 | Central Nervous System Sensitization | No GBD |
| C0221500 | Effects of heat | No GBD | C2982732 | Non-Neoplastic Pediatric Disorder | No GBD |
| C0231218 | Malaise | No GBD | C3163620 | Hypotension Adverse Event | No GBD |
| C0231239 | Fluctuation | No GBD | C3178781 | Vascular Stiffness | No GBD |
| C0231617 | Catch - Finding of sensory dimension of pain | No GBD | C3203359 | Rupture | No GBD |
| C0231807 | Dyspnea on exertion | No GBD | C3263722 | Traumatic AND/OR non-traumatic injury | No GBD |
| C0232197 | Heart Fibrillation | Cardiovascular and circulatory diseases | C3263723 | Traumatic injury | No GBD |
| C0233494 | Tension | No GBD | C3272363 | Ischemic Cerebrovascular Accident | Cardiovascular and circulatory diseases |
| C0233601 | Spraying behavior | No GBD | C3463824 | MYELODYSPLASTIC SYNDROME | No GBD |
| C0233629 | Thinking and speaking disturbances | No GBD | C3489393 | Hiatal Hernia | No GBD |
| C0233762 | Auditory hallucinations | No GBD | C3496054 | Cerebral Palsy, Ataxic, Autosomal Recessive | No GBD |
| C0233794 | Memory impairment | No GBD | C3539781 | Progressive cGVHD | Injuries |
| C0234215 | Sensory Discomfort | No GBD | C3539909 | Allergic disposition | No GBD |
| C0234233 | Sore to touch | No GBD | C3542022 | SHORT STATURE, ONYCHODYSPLASIA, FACIAL DYSMORPHISM, AND HYPOTRICHOSIS SYNDROME | No GBD |
| C0234253 | Rest pain | No GBD | C3542024 | AORTIC VALVE DISEASE 2 | Cardiovascular and circulatory diseases |
| C0235063 | Respiratory Depression | No GBD | C3543005 | Surgical incision wound (morphologic abnormality) | No GBD |
| C0235169 | Excitability | No GBD | C3665444 | Neutrophilia (disorder) | No GBD |
| C0235394 | Wasting | No GBD | C3668885 | Cuffing (morphologic abnormality) | No GBD |
| C0235527 | Heart Failure, Right-Sided | No GBD | C3714514 | Infection | No GBD |
| C0236865 | Relational problem NOS | No GBD | C3714552 | Weakness | No GBD |
| C0236969 | Substance-Related Disorders | No GBD | C3714660 | Trauma | No GBD |
| C0237123 | Alcohol or Other Drugs use | Mental and behavioral disorders | C3811653 | Experimental Organism Basal Cell Carcinoma | Neoplasms |
| C0237593 | Emotionality | No GBD | C3811884 | BRAF V600 Mutation | No GBD |
| C0238217 | Renal transplant rejection | No GBD | C3812172 | BRAF V600 Protein Variation | No GBD |
| C0238218 | Tear of meniscus of knee | No GBD | C3825414 | Pain in children | No GBD |
| C0239313 | exercise induced | No GBD |  |  |  |

**Dataset S2: Expert-based enrichment database for the classification to the 171 GBD categories.**

| CUI | Concept_name | GBD_classification | CUI | Concept_name | GBD_classification |
| --- | --- | --- | --- | --- | --- |
| C0000737 | Abdominal Pain | No GBD | C0235394 | Wasting | No GBD |
| C0000809 | Abortion, Habitual | Maternal disorders | C0235527 | Heart Failure, Right-Sided | No GBD |
| C0000924 | Accidents | No GBD | C0236865 | Relational problem NOS | No GBD |
| C0000925 | Incised wound | No GBD | C0236969 | Substance-Related Disorders | No GBD |
| C0001122 | Acidosis | No GBD | C0237123 | Alcohol or Other Drugs use | Alcohol use disorders AND Drug use disorders |
| C0001125 | Acidosis, Lactic | No GBD | C0237593 | Emotionality | No GBD |
| C0001314 | Acute Disease | No GBD | C0238198 | Gastrointestinal Stromal Tumors | Bladder cancer AND Brain and nervous system cancers AND Breast cancer AND Cancer of other part of pharynx and oropharynx AND Cervical cancer AND Colon and rectum cancers AND Esophageal cancer AND Gallbladder and biliary tract cancer AND Hodgkin's disease AND Kidney and other urinary organ cancers AND Larynx cancer AND Leukemia AND Liver cancer AND Malignant melanoma of skin AND Mouth cancer AND Multiple myeloma AND Nasopharynx cancer AND Non-Hodgkin lymphoma AND Non-melanoma skin cancer AND Ovarian cancer AND Pancreatic cancer AND Prostate cancer AND Stomach cancer AND Testicular cancer AND Thyroid cancer AND Trachea, bronchus, and lung cancers AND Uterine cancer |
| C0001511 | Tissue Adhesions | No GBD | C0238217 | Renal transplant rejection | No GBD |
| C0002351 | Altitude Sickness | No GBD | C0238218 | Tear of meniscus of knee | No GBD |
| C0002690 | Amputation Stumps | No GBD | C0239313 | exercise induced | No GBD |
| C0002726 | Amyloidosis | No GBD | C0239833 | Hand pain | No GBD |
| C0002792 | anaphylaxis | No GBD | C0240066 | Iron deficiency | Iron-deficiency anemia |
| C0002874 | Aplastic Anemia | No GBD | C0242184 | Hypoxia | No GBD |
| C0002994 | Angioedema | No GBD | C0242339 | Dyslipidemias | No GBD |
| C0003123 | Anorexia | No GBD | C0242350 | Erectile dysfunction | No GBD |
| C0003578 | Apnea | No GBD | C0242429 | Sore Throat | No GBD |
| C0003782 | Argyria | No GBD | C0242488 | Acute Lung Injury | No GBD |
| C0003794 | Arm Injuries | No GBD | C0242510 | Drug usage | Drug use disorders |
| C0003850 | Arteriosclerosis | No GBD | C0242606 | Oxidative Stress | No GBD |
| C0003864 | Arthritis | No GBD | C0242781 | disease transmission | No GBD |
| C0004144 | Atelectasis | No GBD | C0242966 | Systemic Inflammatory Response Syndrome | No GBD |
| C0004368 | Autoimmunity | No GBD | C0242992 | Multiple Chemical Sensitivity | No GBD |
| C0004606 | Nonproliferative diabetic retinopathy | No GBD | C0243001 | Abdominal Abscess | No GBD |
| C0004610 | Bacteremia | No GBD | C0243026 | Sepsis | No GBD |
| C0004623 | Bacterial Infections | No GBD | C0243083 | associated disease | No GBD |
| C0004930 | Behavior Disorders | No GBD | C0243088 | sequelae aspects | No GBD |
| C0004936 | Mental disorders | No GBD | C0262593 | Peripheral Nerve Injuries | No GBD |
| C0005658 | bite injury | No GBD | C0262627 | Seroma | No GBD |
| C0005741 | Blepharitis | No GBD | C0264490 | Acute respiratory failure | No GBD |
| C0006107 | Brain Concussion | No GBD | C0264492 | Chronic respiratory failure | No GBD |
| C0006114 | Cerebral Edema | No GBD | C0264886 | Conduction disorder of the heart | No GBD |
| C0006434 | Burn injury | Fire, heat and hot substances | C0264956 | Atheroma | No GBD |
| C0006625 | Cachexia | No GBD | C0264995 | Occlusion of artery (disorder) | No GBD |
| C0007222 | Cardiovascular Diseases | No GBD | C0266836 | Infantile Colic | No GBD |
| C0007570 | Celiac Disease | No GBD | C0267716 | Incisional hernia | No GBD |
| C0007789 | Cerebral Palsy | No GBD | C0268064 | Transfusion hemosiderosis | No GBD |
| C0008031 | Chest Pain | No GBD | C0268381 | Primary amyloidosis | No GBD |
| C0008039 | Cheyne-Stokes Respiration | No GBD | C0268407 | Senile cardiac amyloidosis | No GBD |
| C0008625 | Chromosome Aberrations | No GBD | C0268575 | Isovaleryl-CoA dehydrogenase deficiency | No GBD |
| C0008679 | Chronic disease | No GBD | C0270075 | Perinatal disorder | No GBD |
| C0008715 | Chronically Ill | No GBD | C0270327 | Bedwetting | No GBD |
| C0008767 | Cicatrization | No GBD | C0270805 | Hemiplegic cerebral palsy | No GBD |
| C0009421 | Comatose | No GBD | C0271051 | Macular retinal edema | No GBD |
| C0009450 | Communicable Diseases | No GBD | C0271641 | Malnutrition-related diabetes mellitus | Diabetes mellitus |
| C0009492 | Compartment syndromes | No GBD | C0271650 | Impaired glucose tolerance | Diabetes mellitus |
| C0009566 | Complication | No GBD | C0274281 | Injury due to exposure to external cause | No GBD |
| C0009814 | Acquired stenosis | No GBD | C0275551 | Primary bacterial peritonitis | No GBD |
| C0009938 | Contusions | No GBD | C0277556 | Recurrent disease | No GBD |
| C0010046 | Corn of toe | No GBD | C0277562 | Adult disease | No GBD |
| C0010068 | Coronary heart disease | Ischemic heart disease | C0277564 | Acquired disease | No GBD |
| C0010200 | Coughing | No GBD | C0278139 | Moderate pain | No GBD |
| C0010340 | Critical Illness | No GBD | C0278996 | Cancer of Head and Neck | Larynx cancer AND Mouth cancer AND Cancer of other part of pharynx and oropharynx |
| C0010674 | Cystic Fibrosis | No GBD | C0280334 | Paranasal Sinus Squamous Cell Carcinoma | Cancer of other part of pharynx and oropharynx |
| C0010957 | Tissue damage | No GBD | C0280628 | Recurrent Uterine Sarcoma | No GBD |
| C0011164 | Abnormal degeneration | No GBD | C0280630 | Uterine Carcinosarcoma | Uterine cancer |
| C0011168 | Deglutition Disorders | No GBD | C0281856 | Generalized aches and pains | No GBD |
| C0011175 | Dehydration | No GBD | C0282193 | Iron Overload | No GBD |
| C0011389 | Dental Plaque | Periodontal disease | C0282666 | Very Low Birth Weight | No GBD |
| C0011560 | Amyloid deposition | No GBD | C0314719 | Dryness of eye | No GBD |
| C0011847 | Diabetes | Diabetes mellitus | C0332448 | Infiltration | No GBD |
| C0011849 | Diabetes Mellitus | Diabetes mellitus | C0332568 | Pad Mass | No GBD |
| C0011854 | Diabetes Mellitus, Insulin-Dependent | Diabetes mellitus | C0332679 | Crushing injury | Road injury AND Exposure to mechanical forces |
| C0011860 | Diabetes Mellitus, Non-Insulin-Dependent | Diabetes mellitus | C0332687 | Blisters, epidermal loss [second degree], unspecified site | No GBD |
| C0011880 | Diabetic Ketoacidosis | Diabetes mellitus | C0332798 | Open wound | No GBD |
| C0011881 | Diabetic Nephropathy | Diabetes mellitus | C0332803 | Surgical wound | No GBD |
| C0011882 | Diabetic Neuropathies | Diabetes mellitus | C0332837 | Traumatic implants | No GBD |
| C0011884 | Diabetic Retinopathy | No GBD | C0332840 | Amputated structure (morphologic abnormality) | No GBD |
| C0011991 | Diarrhea | No GBD | C0332853 | Anastomosis | No GBD |
| C0012634 | Disease | No GBD | C0333186 | Restenosis | Ischemic heart disease |
| C0012739 | Disseminated Intravascular Coagulation | No GBD | C0333262 | Vesicle (morphologic abnormality) | No GBD |
| C0012833 | Dizziness | No GBD | C0333641 | Atrophic | No GBD |
| C0013146 | Drug abuse | Drug use disorders | C0334094 | Proliferation (morphologic abnormality) | No GBD |
| C0013312 | Dupuytren Contracture | No GBD | C0334299 | Carcinoid tumor no ICD-O subtype | Bladder cancer AND Brain and nervous system cancers AND Breast cancer AND Cancer of other part of pharynx and oropharynx AND Cervical cancer AND Colon and rectum cancers AND Esophageal cancer AND Gallbladder and biliary tract cancer AND Hodgkin's disease AND Kidney and other urinary organ cancers AND Larynx cancer AND Leukemia AND Liver cancer AND Malignant melanoma of skin AND Mouth cancer AND Multiple myeloma AND Nasopharynx cancer AND Non-Hodgkin lymphoma AND Non-melanoma skin cancer AND Ovarian cancer AND Pancreatic cancer AND Prostate cancer AND Stomach cancer AND Testicular cancer AND Thyroid cancer AND Trachea, bronchus, and lung cancers AND Uterine cancer |
| C0013404 | Dyspnea | No GBD | C0338596 | Spastic cerebral palsy | No GBD |
| C0013595 | Eczema | Eczema | C0338656 | Impaired cognition | No GBD |
| C0013604 | Edema | No GBD | C0339546 | Retinal Pigment Epithelial Detachment | No GBD |
| C0013687 | effusion | No GBD | C0340861 | Electromechanical dissociation | No GBD |
| C0013922 | Embolism | No GBD | C0341539 | Parastomal hernia NOS | No GBD |
| C0014009 | Empyema | No GBD | C0342257 | Complications of Diabetes Mellitus | Diabetes mellitus |
| C0014236 | Endophthalmitis | No GBD | C0342289 | Diabetes-deafness syndrome maternally transmitted (disorder) | Diabetes mellitus |
| C0014518 | Toxic Epidermal Necrolysis | No GBD | C0342751 | Generalized glycogen storage disease of infants | No GBD |
| C0014591 | Epistaxis | No GBD | C0342895 | Fish-Eye Disease | No GBD |
| C0014742 | Erythema Multiforme | No GBD | C0343641 | Human papilloma virus infection | No GBD |
| C0015230 | Exanthema | No GBD | C0344307 | Absence of pain sensation | No GBD |
| C0015376 | Extravasation | No GBD | C0346153 | Breast Cancer, Familial | Breast cancer |
| C0015625 | Fanconi Anemia | No GBD | C0346627 | Intestinal Cancer | No GBD |
| C0015672 | Fatigue | No GBD | C0346975 | Secondary malignant neoplasm of rectum | No GBD |
| C0015674 | Chronic Fatigue Syndrome | No GBD | C0348921 | Pre-existing diabetes mellitus, non-insulin-dependent | Diabetes mellitus |
| C0015732 | Fecal Incontinence | No GBD | C0362046 | Prediabetes syndrome | Diabetes mellitus |
| C0015802 | Fracture of femur, part unspecified | No GBD | C0376297 | Cardiac Death | No GBD |
| C0015806 | Fracture of neck of femur | No GBD | C0392164 | Pulmonary Cystic Fibrosis | No GBD |
| C0016053 | Fibromyalgia | No GBD | C0392615 | Adverse effect of radiation therapy | Adverse effects of medical treatment |
| C0016059 | Fibrosis | No GBD | C0392618 | Postoperative infection | No GBD |
| C0016169 | pathologic fistula | No GBD | C0392707 | Atopy | No GBD |
| C0016204 | Flatulence | No GBD | C0398353 | Hypercapnic respiratory failure | No GBD |
| C0016382 | Flushing | No GBD | C0398650 | Immune thrombocytopenic purpura | No GBD |
| C0016385 | Cardiac Flutter | Atrial fibrillation and flutter | C0403447 | Chronic Kidney Insufficiency | Chronic kidney diseases |
| C0016470 | Food Allergy | No GBD | C0409312 | Rupture of anterior cruciate ligament | No GBD |
| C0016658 | Fracture | No GBD | C0410158 | Muscle damage | No GBD |
| C0016662 | Fracture of unspecified bone, open | No GBD | C0410256 | muscle injury | No GBD |
| C0017168 | Gastroesophageal reflux disease | No GBD | C0413252 | Hypothermia due to exposure | No GBD |
| C0017181 | Gastrointestinal hemorrhage, unspecified | No GBD | C0426070 | Flexed fetal attitude | No GBD |
| C0018133 | Graft-vs-Host Disease | Adverse effects of medical treatment | C0427149 | Gait, Drop Foot | No GBD |
| C0018273 | Growth Disorders | No GBD | C0428908 | Sinus Node Dysfunction (disorder) | No GBD |
| C0018482 | Haemophilus Infections | No GBD | C0428977 | Bradycardia | No GBD |
| C0018520 | Halitosis | No GBD | C0439857 | Dependence | No GBD |
| C0018674 | Unspecified injury of head | No GBD | C0442874 | Neuropathy | No GBD |
| C0018681 | Headache | No GBD | C0443146 | Autoimmune reaction | No GBD |
| C0018790 | Cardiac Arrest | No GBD | C0443306 | Spastic | No GBD |
| C0018799 | Heart Diseases | No GBD | C0449430 | Physiological Stress | No GBD |
| C0018801 | Heart failure | No GBD | C0452131 | Effects of vibration | No GBD |
| C0018802 | Congestive heart failure | No GBD | C0474368 | Labor Pain | No GBD |
| C0018834 | Heartburn | No GBD | C0476273 | Respiratory distress | No GBD |
| C0018944 | Hematoma | No GBD | C0481391 | Lifestyle-related condition | No GBD |
| C0018991 | Hemiplegia, unspecified | No GBD | C0494165 | Secondary malignant neoplasm of liver | No GBD |
| C0019054 | Hemolysis (disorder) | No GBD | C0496118 | Effects of electric current | No GBD |
| C0019080 | Hemorrhage | No GBD | C0497406 | Overweight | No GBD |
| C0019247 | Hereditary Diseases | No GBD | C0520679 | Sleep Apnea, Obstructive | No GBD |
| C0019322 | Umbilical hernia | No GBD | C0520817 | Physical disability | No GBD |
| C0019326 | Ventral Hernia | No GBD | C0520893 | Localized desquamation | No GBD |
| C0019557 | Hip Fractures | No GBD | C0520904 | Postoperative Nausea | No GBD |
| C0020162 | Fracture of humerus | No GBD | C0520905 | Vomiting, Postoperative | No GBD |
| C0020175 | Hunger | No GBD | C0520909 | Postoperative Nausea and Vomiting | No GBD |
| C0020429 | Hyperalgesia | No GBD | C0524528 | Pervasive Development Disorder | Asperger's syndrome AND Autism |
| C0020443 | Hypercholesterolemia | No GBD | C0524620 | Metabolic Syndrome X | No GBD |
| C0020456 | Hyperglycemia | Diabetes mellitus | C0544885 | Mutation, Nonsense | No GBD |
| C0020459 | Hyperinsulinism | No GBD | C0546255 | Colostomy site | No GBD |
| C0020488 | Hypernatremia | No GBD | C0546256 | Gastrostomy site | No GBD |
| C0020505 | Hyperphagia | No GBD | C0546817 | Fluid overload | No GBD |
| C0020507 | Hyperplasia | No GBD | C0559307 | Breathy voice quality | No GBD |
| C0020517 | Hypersensitivity | No GBD | C0559469 | Allergy to eggs | No GBD |
| C0020523 | Immediate hypersensitivity | No GBD | C0559470 | Allergy to peanuts | No GBD |
| C0020542 | Pulmonary Hypertension | No GBD | C0574785 | Lower Urinary Tract Symptoms | No GBD |
| C0020564 | Hypertrophy | No GBD | C0575090 | Equilibration disorder | No GBD |
| C0020615 | Hypoglycemia | No GBD | C0577573 | Mass of body region | No GBD |
| C0020963 | Immune Tolerance | No GBD | C0595921 | Intraocular pressure disorder | Glaucoma |
| C0021053 | Immune System Diseases | No GBD | C0596988 | mutant | No GBD |
| C0021167 | Incontinence | No GBD | C0599156 | Transition Mutation | No GBD |
| C0021290 | Neonatal disorder | Preterm birth complications AND Sepsis and other infectious disorders of the newborn baby | C0599766 | Recovery of Function | No GBD |
| C0021368 | Inflammation | No GBD | C0600142 | Hot flushes | No GBD |
| C0021655 | Insulin Resistance | Diabetes mellitus | C0600518 | Choroidal Neovascularization | No GBD |
| C0022104 | Irritable Bowel Syndrome | No GBD | C0600688 | Toxic effect | No GBD |
| C0022346 | Icterus | No GBD | C0677660 | Emotional problems | No GBD |
| C0022602 | Actinic keratosis | No GBD | C0677932 | Progressive Neoplastic Disease | Bladder cancer AND Brain and nervous system cancers AND Breast cancer AND Cancer of other part of pharynx and oropharynx AND Cervical cancer AND Colon and rectum cancers AND Esophageal cancer AND Gallbladder and biliary tract cancer AND Hodgkin's disease AND Kidney and other urinary organ cancers AND Larynx cancer AND Leukemia AND Liver cancer AND Malignant melanoma of skin AND Mouth cancer AND Multiple myeloma AND Nasopharynx cancer AND Non-Hodgkin lymphoma AND Non-melanoma skin cancer AND Ovarian cancer AND Pancreatic cancer AND Prostate cancer AND Stomach cancer AND Testicular cancer AND Thyroid cancer AND Trachea, bronchus, and lung cancers AND Uterine cancer |
| C0022660 | Kidney Failure, Acute | No GBD | C0678236 | Rare Diseases | No GBD |
| C0022661 | Kidney Failure, Chronic | Chronic kidney diseases | C0678356 | alcohol effect | No GBD |
| C0022744 | Knee Injuries | No GBD | C0679381 | Pediatric Disorder | No GBD |
| C0022951 | Lactose Intolerance | No GBD | C0679861 | Complications of treatment | Adverse effects of medical treatment |
| C0023212 | Left-Sided Heart Failure | No GBD | C0683278 | Mental Suffering | No GBD |
| C0023530 | Leukopenia | No GBD | C0684309 | Disease model | No GBD |
| C0024141 | Lupus Erythematosus, Systemic | No GBD | C0684320 | Disease regression | No GBD |
| C0024440 | Macular Edema, Cystoid | No GBD | C0684336 | Impaired health | No GBD |
| C0026771 | Unspecified multiple injuries | No GBD | C0685938 | Malignant neoplasm of gastrointestinal tract | Esophageal cancer AND Stomach cancer AND Colon and rectum cancers |
| C0026821 | Muscle Cramp | No GBD | C0686377 | CNS metastases | No GBD |
| C0026837 | Muscle Rigidity | No GBD | C0686619 | Secondary and unspecified malignant neoplasm of lymph node, unspecified | No GBD |
| C0026838 | Muscle Spasticity | No GBD | C0694563 | Excessive daytime somnolence | No GBD |
| C0026986 | Dysmyelopoietic Syndromes | No GBD | C0699753 | Cancer Relapse | No GBD |
| C0027497 | Nausea | No GBD | C0700198 | Pulmonary aspiration | No GBD |
| C0027498 | Nausea and vomiting | No GBD | C0700319 | Mentally ill chemical abuse | Drug use disorders |
| C0027769 | Nervousness | No GBD | C0700323 | Observation of Neuromuscular Block | No GBD |
| C0027796 | Neuralgia | No GBD | C0700361 | Emotional distress | No GBD |
| C0027947 | Neutropenia | No GBD | C0728867 | Drug effect disorder | Drug use disorders |
| C0027960 | Nevus | No GBD | C0730285 | Diabetic macular edema | Diabetes mellitus |
| C0028043 | Nicotine Dependence | No GBD | C0730345 | Microalbuminuria | No GBD |
| C0028259 | Nodule | No GBD | C0740697 | Psychosocial problem | No GBD |
| C0028734 | Nocturia | No GBD | C0741282 | atrial fibrillation recurrent | Atrial fibrillation and flutter |
| C0028754 | Obesity | No GBD | C0741923 | cardiac event | No GBD |
| C0028778 | Obstruction | No GBD | C0742006 | Catheter infection | No GBD |
| C0029443 | Osteomyelitis | No GBD | C0746787 | Cancer of Neck | Larynx cancer AND Mouth cancer AND Cancer of other part of pharynx and oropharynx |
| C0029453 | Osteopenia | No GBD | C0751177 | Cancer of Head | Larynx cancer AND Mouth cancer AND Cancer of other part of pharynx and oropharynx |
| C0029456 | Osteoporosis | No GBD | C0751956 | Acute Cerebrovascular Accidents | Cerebrovascular disease |
| C0029458 | Osteoporosis, Postmenopausal | No GBD | C0815107 | psychological distress | Anxiety disorders |
| C0029944 | Drug Overdose | Drug use disorders | C0847097 | Heartburn acidity | No GBD |
| C0030193 | Pain | No GBD | C0848377 | trauma to the abdomen | No GBD |
| C0030200 | Pain, Intractable | No GBD | C0850624 | cardiovascular risk factor | No GBD |
| C0030201 | Pain, Postoperative | No GBD | C0850758 | Pelvic pain female | No GBD |
| C0030486 | Paraplegia, unspecified | No GBD | C0852694 | Cartilage injury | No GBD |
| C0030499 | Parasitic Diseases | No GBD | C0854135 | Pseudomonas aeruginosa infection | No GBD |
| C0030794 | Pelvic Pain | No GBD | C0854706 | Infection of newborn NOS | Sepsis and other infectious disorders of the newborn baby |
| C0031117 | Peripheral Neuropathy | No GBD | C0856169 | endothelial dysfunction | No GBD |
| C0031154 | Peritonitis, unspecified | No GBD | C0857122 | hyponatremic | No GBD |
| C0031925 | Pilonidal Cyst | No GBD | C0857496 | Thromboembolic event | No GBD |
| C0032227 | Pleural effusion disorder | No GBD | C0860239 | Catheter related infection | No GBD |
| C0032320 | Pneumoperitoneum | No GBD | C0866323 | Severe toxemia | No GBD |
| C0032326 | Pneumothorax | No GBD | C0867389 | Chronic graft-versus-host disease | Adverse effects of medical treatment |
| C0032343 | Poisoning | Poisonings | C0876926 | Traumatic Brain Injury | No GBD |
| C0032463 | Polycythemia Vera | No GBD | C0877008 | Enzyme inhibition disorder | No GBD |
| C0032787 | Postoperative Complications | No GBD | C0877248 | Adverse event | No GBD |
| C0032788 | Postoperative Hemorrhage | No GBD | C0877445 | Candidemia | No GBD |
| C0032962 | Pregnancy Complications | Maternal disorders | C0878787 | Growth failure | No GBD |
| C0033027 | Preleukemia | No GBD | C0879626 | Adverse effects | No GBD |
| C0033038 | Premature Ejaculation | No GBD | C0917801 | Sleeplessness | No GBD |
| C0033119 | Puncture wound | No GBD | C0920269 | Microsatellite Instability | No GBD |
| C0033139 | Primary Insomnia | No GBD | C0920563 | Insulin Sensitivity | No GBD |
| C0034063 | Pulmonary Edema | No GBD | C0940933 | infection as a complication | No GBD |
| C0034065 | Pulmonary Embolism | No GBD | C0948008 | Ischemic stroke | Cerebrovascular disease |
| C0034372 | Quadriplegia | No GBD | C0949177 | White spot lesion | No GBD |
| C0034535 | Radiation Syndrome | Adverse effects of medical treatment | C1096106 | wound complication | No GBD |
| C0035067 | Renal Artery Stenosis | No GBD | C1110554 | Cardiovascular occlusion | Ischemic heart disease |
| C0035078 | Kidney Failure | No GBD | C1112442 | Female sexual dysfunction | No GBD |
| C0035222 | Respiratory Distress Syndrome, Adult | No GBD | C1135191 | Unspecified systolic (congestive) heart failure | No GBD |
| C0035229 | Respiratory Insufficiency | No GBD | C1135196 | Unspecified diastolic (congestive) heart failure | No GBD |
| C0035242 | Respiratory Tract Diseases | No GBD | C1135361 | Persistent pulmonary hypertension | No GBD |
| C0035328 | Retinal Vein Occlusion | Cerebrovascular disease | C1140999 | Contraction (finding) | No GBD |
| C0035455 | Rhinitis | No GBD | C1142264 | critical limb ischemia | No GBD |
| C0036421 | Systemic Scleroderma | No GBD | C1145670 | Respiratory Failure | No GBD |
| C0036429 | Sclerosis | No GBD | C1155266 | inflammatory response | No GBD |
| C0036572 | Seizures | No GBD | C1171258 | Complication Aspects | No GBD |
| C0036690 | Septicemia | No GBD | C1260899 | Anemia, Diamond-Blackfan | No GBD |
| C0036877 | organic sexual dysfunction | No GBD | C1260922 | Unspecified abnormalities of breathing | No GBD |
| C0036974 | Shock | No GBD | C1261287 | Stenosis | No GBD |
| C0036980 | Cardiogenic shock | No GBD | C1261473 | Sarcoma | Bladder cancer AND Brain and nervous system cancers AND Breast cancer AND Cancer of other part of pharynx and oropharynx AND Cervical cancer AND Colon and rectum cancers AND Esophageal cancer AND Gallbladder and biliary tract cancer AND Hodgkin's disease AND Kidney and other urinary organ cancers AND Larynx cancer AND Leukemia AND Liver cancer AND Malignant melanoma of skin AND Mouth cancer AND Multiple myeloma AND Nasopharynx cancer AND Non-Hodgkin lymphoma AND Non-melanoma skin cancer AND Ovarian cancer AND Pancreatic cancer AND Prostate cancer AND Stomach cancer AND Testicular cancer AND Thyroid cancer AND Trachea, bronchus, and lung cancers AND Uterine cancer |
| C0036982 | Shock, Hemorrhagic | No GBD | C1262018 | Graft failure | No GBD |
| C0036983 | Septic Shock | No GBD | C1265601 | Solitary mass | No GBD |
| C0036992 | Short Bowel Syndrome | Adverse effects of medical treatment | C1265679 | Wound, healed (morphologic abnormality) | No GBD |
| C0037006 | Shoulder Fractures | No GBD | C1265875 | Disintegration (morphologic abnormality) | No GBD |
| C0037011 | Shoulder Pain | No GBD | C1270972 | Mild cognitive disorder | No GBD |
| C0037052 | Sick Sinus Syndrome | No GBD | C1275684 | Meibomian gland dysfunction | No GBD |
| C0037088 | Signs and Symptoms | No GBD | C1277187 | Left ventricular systolic dysfunction | No GBD |
| C0037315 | Sleep Apnea Syndromes | No GBD | C1291077 | Abdominal bloating | No GBD |
| C0037384 | Snoring | No GBD | C1298685 | Chronic pain syndrome | No GBD |
| C0037578 | Soft Tissue Injuries | No GBD | C1306597 | Psychiatric problem | No GBD |
| C0037763 | Spasm | No GBD | C1313952 | Periodic breathing | No GBD |
| C0037929 | Spinal Cord Injuries | No GBD | C1368999 | Late effect of medical intervention | Adverse effects of medical treatment |
| C0038443 | Stress, Psychological | No GBD | C1373218 | Decreased Immunologic Activity [PE] | No GBD |
| C0038454 | Cerebrovascular accident | Cerebrovascular disease | C1384600 | Systemic onset juvenile chronic arthritis | No GBD |
| C0038561 | Submersion | No GBD | C1392786 | Alteration Of Cognitive Function | No GBD |
| C0038580 | Substance Dependence | Drug use disorders AND Alcohol use disorders | C1397014 | Imbalance | No GBD |
| C0038586 | Substance Use Disorders | Drug use disorders AND Alcohol use disorders | C1442161 | Gene Deletion Abnormality | No GBD |
| C0038587 | Substance Withdrawal Syndrome | Drug use disorders AND Alcohol use disorders AND Adverse effects of medical treatment | C1456781 | Benign melanocytic nevus | No GBD |
| C0038941 | Surgical Wound Infection | No GBD | C1457887 | Symptoms | No GBD |
| C0039070 | Syncope | No GBD | C1504525 | Acute lymphoblastic leukemia recurrent | Leukemia |
| C0039082 | Syndrome | No GBD | C1510420 | Cavitation | No GBD |
| C0039231 | Tachycardia | No GBD | C1510432 | Radiation Sickness | Adverse effects of medical treatment |
| C0039504 | Tendon Injuries | No GBD | C1510586 | Autism Spectrum Disorders | Autism |
| C0040185 | Tibial Fractures | No GBD | C1512441 | High-Risk Cancer | No GBD |
| C0040456 | Impacted tooth | No GBD | C1516986 | Evaluable Disease | No GBD |
| C0040822 | Tremor | No GBD | C1517205 | Flare | No GBD |
| C0041755 | Adverse reaction to drug | Adverse effects of medical treatment | C1521461 | Loss of Chromosome 5q | No GBD |
| C0041834 | Erythema | No GBD | C1521724 | Alzheimer's Disease Pathway KEGG | Alzheimer's disease and other dementias |
| C0041909 | Upper gastrointestinal hemorrhage | No GBD | C1527304 | Allergic Reaction | No GBD |
| C0042024 | Urinary Incontinence | No GBD | C1527311 | Brain Edema | No GBD |
| C0042025 | Urinary Stress Incontinence | No GBD | C1533163 | Disorder of cellular component of blood | No GBD |
| C0042164 | Uveitis | No GBD | C1535939 | Pneumocystis jiroveci pneumonia | No GBD |
| C0042373 | Vascular Diseases | Aortic aneurysm AND Atrial fibrillation and flutter AND Cardiomyopathy and myocarditis AND Cerebrovascular disease AND Endocarditis AND Hypertensive heart disease AND Ischemic heart disease AND Peripheral vascular disease AND Rheumatic heart disease | C1559154 | Rash and Dermatitis Adverse Event Associated with Chemoradiation | Adverse effects of medical treatment |
| C0042510 | Ventricular Fibrillation | No GBD | C1561642 | Chronic kidney disease, Stage V | Chronic kidney diseases |
| C0042571 | Vertigo | No GBD | C1561643 | Chronic Kidney Diseases | Chronic kidney diseases |
| C0042749 | Viremia, unspecified | No GBD | C1565249 | Mobility Limitation | No GBD |
| C0042870 | Vitamin D Deficiency | No GBD | C1565489 | Renal Insufficiency | Chronic kidney diseases |
| C0042963 | Vomiting | No GBD | C1566590 | Delayed Graft Function | No GBD |
| C0043144 | Wheezing | No GBD | C1609535 | Invasive Candidiasis | No GBD |
| C0043145 | Whiplash Injuries | Interpersonal violence | C1704376 | Uterine Corpus Carcinosarcoma | Uterine cancer |
| C0043194 | Wiskott-Aldrich Syndrome | No GBD | C1705285 | Mutation Abnormality | No GBD |
| C0043241 | Wound Infection | No GBD | C1705427 | Germline Mutation Abnormality | No GBD |
| C0043246 | Laceration | No GBD | C1705759 | Gene Amplification Abnormality | No GBD |
| C0043250 | Injury wounds | No GBD | C1709246 | Non-Neoplastic Disorder | No GBD |
| C0043251 | Wounds and Injuries | No GBD | C1719672 | Severe Sepsis | No GBD |
| C0043352 | Xerostomia | No GBD | C1832387 | DIABETES MELLITUS, NONINSULIN-DEPENDENT, 2 (disorder) | Diabetes mellitus |
| C0079487 | Helicobacter Infections | Gastritis and duodenitis | C1832544 | DIABETES MELLITUS, NONINSULIN-DEPENDENT, 1 (disorder) | Diabetes mellitus |
| C0080032 | Malignant pleural effusion | No GBD | C1838069 | SCHIZOPHRENIA 3 (disorder) | Schizophrenia |
| C0080194 | Muscle strain | No GBD | C1838261 | DIABETES MELLITUS, INSULIN-DEPENDENT, 4 | Diabetes mellitus |
| C0085083 | Ovarian Hyperstimulation Syndrome | No GBD | C1838262 | DIABETES MELLITUS, INSULIN-DEPENDENT, 3 | Diabetes mellitus |
| C0085111 | Ankle Injuries | No GBD | C1842642 | Diabetes Mellitus, Noninsulin-Dependent, Type 4 | Diabetes mellitus |
| C0085281 | Addictive Behavior | Alcohol use disorders AND Drug use disorders | C1847835 | VITILIGO-ASSOCIATED MULTIPLE AUTOIMMUNE DISEASE SUSCEPTIBILITY 1 (finding) | No GBD |
| C0085298 | Sudden Cardiac Death | No GBD | C1848140 | COGNITIVE FUNCTION 1, SOCIAL | No GBD |
| C0085426 | Gram-Positive Bacterial Infections | No GBD | C1850383 | Neuropathy, Painful | No GBD |
| C0085580 | Essential Hypertension | No GBD | C1852092 | DIABETES MELLITUS, INSULIN-DEPENDENT, 2 | Diabetes mellitus |
| C0085584 | Encephalopathies | No GBD | C1860404 | VITREORETINOPATHY, NEOVASCULAR INFLAMMATORY | No GBD |
| C0085612 | Ventricular arrhythmia | No GBD | C1863594 | Diabetes Mellitus, Noninsulin-Dependent, 3 | Diabetes mellitus |
| C0085631 | Agitation | No GBD | C1864068 | DIABETES MELLITUS, INSULIN-DEPENDENT, 17 | Diabetes mellitus |
| C0085632 | Indifferent mood | No GBD | C1866041 | DIABETES MELLITUS, INSULIN-DEPENDENT, 6 | Diabetes mellitus |
| C0085633 | Mood swings | Bipolar affective disorder | C1868193 | PNEUMOTHORAX, PRIMARY SPONTANEOUS | No GBD |
| C0086439 | Hypokinesia | No GBD | C1868649 | PANIC DISORDER 1 | Anxiety disorders |
| C0087086 | Thrombus | No GBD | C1883018 | Severe Aplastic Anemia | No GBD |
| C0087169 | Withdrawal Symptoms | Drug use disorders AND Alcohol use disorders AND Adverse effects of medical treatment | C1959609 | Erosion lesion | No GBD |
| C0149721 | Left Ventricular Hypertrophy | No GBD | C1962948 | Atelectasis Adverse Event | No GBD |
| C0149746 | Orthostasis | No GBD | C1963065 | Apnea Adverse Event | No GBD |
| C0149871 | Deep Vein Thrombosis | No GBD | C1963179 | Nausea Adverse Event | No GBD |
| C0150055 | Chronic pain | No GBD | C1998242 | Traumatic injury of skeletal muscle | No GBD |
| C0151293 | Chronic Headache | Tension-type headache AND Migraine | C2004489 | Regurgitation | No GBD |
| C0151517 | Complete atrioventricular block | No GBD | C2004491 | Cicatrix | No GBD |
| C0151740 | Intracranial Hypertension | No GBD | C2316810 | Chronic kidney disease stage 5 | Chronic kidney diseases |
| C0152020 | Gastroparesis | No GBD | C2349423 | Medication overuse headache | No GBD |
| C0152171 | Idiopathic pulmonary hypertension | No GBD | C2586211 | Thrombosis of blood vessel | No GBD |
| C0153567 | Uterine Cancer | Uterine cancer | C2607914 | Allergic rhinitis (disorder) | No GBD |
| C0153661 | Malignant neoplasm of thorax | No GBD | C2609414 | Acute kidney injury | No GBD |
| C0153662 | Malignant neoplasm of abdomen | No GBD | C2712889 | Novel H1N1 influenza | No GBD |
| C0153676 | Secondary malignant neoplasm of lung | No GBD | C2729169 | Wrinkled structure | No GBD |
| C0153687 | Secondary malignant neoplasm of skin | No GBD | C2745965 | Emergencies [Disease/Finding] | No GBD |
| C0153690 | Secondary malignant neoplasm of bone and bone marrow | No GBD | C2825055 | Recurrence (disease attribute) | No GBD |
| C0154183 | Diabetes with other specified manifestations | Diabetes mellitus | C2874046 | Type 1 diabetes mellitus with diabetic neuralgia | Diabetes mellitus |
| C0154830 | Proliferative diabetic retinopathy | No GBD | C2874076 | Type 2 diabetes mellitus with diabetic nephropathy | Diabetes mellitus |
| C0154841 | Central retinal vein occlusion | Cerebrovascular disease | C2882221 | Acute pulmonary embolism NOS | No GBD |
| C0155773 | Portal vein thrombosis | No GBD | C2891275 | Cardiac allograft vasculopathy | No GBD |
| C0155927 | Excessive attrition of teeth | No GBD | C2919062 | Shot (injury) | Interpersonal violence |
| C0156409 | Postmenopausal atrophic vaginitis | No GBD | C2936258 | Peri-Implantitis | Periodontal disease |
| C0159658 | Fracture of clavicle | No GBD | C2936380 | Neointima | No GBD |
| C0159877 | Fracture of ankle | No GBD | C2936490 | Out-of-Hospital Cardiac Arrest | No GBD |
| C0160087 | Sprain of ankle, unspecified site | No GBD | C2938905 | Central Nervous System Sensitization | No GBD |
| C0160420 | Injury of kidney | No GBD | C2982732 | Non-Neoplastic Pediatric Disorder | No GBD |
| C0161479 | Injury of nerve(s) of unspecified body region | No GBD | C3163620 | Hypotension Adverse Event | No GBD |
| C0161816 | Cardiac complications | No GBD | C3178781 | Vascular Stiffness | No GBD |
| C0162566 | Porphyria Cutanea Tarda | No GBD | C3203359 | Rupture | No GBD |
| C0162633 | Viral Shedding | No GBD | C3263722 | Traumatic AND/OR non-traumatic injury | No GBD |
| C0162836 | Hidradenitis Suppurativa | No GBD | C3263723 | Traumatic injury | No GBD |
| C0184567 | Acute onset pain | No GBD | C3272363 | Ischemic Cerebrovascular Accident | Cerebrovascular disease |
| C0220650 | Metastatic malignant neoplasm to brain | No GBD | C3275069 | Chronic Total Occlusion Vessel | No GBD |
| C0220656 | Malignant ascites | No GBD | C3463824 | MYELODYSPLASTIC SYNDROME | No GBD |
| C0220981 | Metabolic acidosis | No GBD | C3469521 | FANCONI ANEMIA, COMPLEMENTATION GROUP A (disorder) | No GBD |
| C0221103 | Visual Suppression | No GBD | C3489393 | Hiatal Hernia | No GBD |
| C0221208 | Traumatic injection site | No GBD | C3495559 | Juvenile arthritis | No GBD |
| C0221500 | Effects of heat | No GBD | C3496054 | Cerebral Palsy, Ataxic, Autosomal Recessive | No GBD |
| C0231218 | Malaise | No GBD | C3539781 | Progressive cGVHD | Adverse effects of medical treatment |
| C0231239 | Fluctuation | No GBD | C3539909 | Allergic disposition | No GBD |
| C0231617 | Catch - Finding of sensory dimension of pain | No GBD | C3542022 | SHORT STATURE, ONYCHODYSPLASIA, FACIAL DYSMORPHISM, AND HYPOTRICHOSIS SYNDROME | No GBD |
| C0231749 | Knee pain | No GBD | C3542024 | AORTIC VALVE DISEASE 2 | Rheumatic heart disease |
| C0231807 | Dyspnea on exertion | No GBD | C3543005 | Surgical incision wound (morphologic abnormality) | No GBD |
| C0232197 | Heart Fibrillation | Atrial fibrillation and flutter | C3665444 | Neutrophilia (disorder) | No GBD |
| C0233494 | Tension | No GBD | C3665593 | Nevus, Epidermal | No GBD |
| C0233601 | Spraying behavior | No GBD | C3668885 | Cuffing (morphologic abnormality) | No GBD |
| C0233629 | Thinking and speaking disturbances | No GBD | C3714514 | Infection | No GBD |
| C0233762 | Auditory hallucinations | No GBD | C3714552 | Weakness | No GBD |
| C0233794 | Memory impairment | No GBD | C3714660 | Trauma | No GBD |
| C0234215 | Sensory Discomfort | No GBD | C3811653 | Experimental Organism Basal Cell Carcinoma | Non-melanoma skin cancer |
| C0234233 | Sore to touch | No GBD | C3811884 | BRAF V600 Mutation | No GBD |
| C0234253 | Rest pain | No GBD | C3812172 | BRAF V600 Protein Variation | No GBD |
| C0235063 | Respiratory Depression | No GBD | C3825414 | Pain in children | No GBD |
| C0235169 | Excitability | No GBD |  |  |  |
